# The quality of veterinary medicines and their implications for One Health

**DOI:** 10.1136/bmjgh-2022-008564

**Published:** 2022-08-01

**Authors:** Vayouly Vidhamaly, Konnie Bellingham, Paul N Newton, Céline Caillet

**Affiliations:** 1 Lao-Oxford-Mahosot Hospital-Wellcome Trust Research Unit, Medicine Quality Research Group, Microbiology Laboratory, Mahosot Hospital, Vientiane, Lao People's Democratic Republic; 2 Nuffield Department of Medicine, Medicine Quality Research Group, University of Oxford Centre for Tropical Medicine and Global Health, Oxford, UK; 3 Nuffield Department of Medicine, Infectious Diseases Data Observatory (IDDO)/WorldWide Antimalarial Resistance Network (WWARN), Medicine Quality Research Group, University of Oxford, Oxford, UK

**Keywords:** epidemiology, public health, systematic review

## Abstract

**Objective:**

Substandard and falsified (SF) veterinary medicines affect animal health, agricultural production and food security and will influence antimicrobial resistance (AMR) in both animals and humans. Yet, our understanding of their extent and impact is poor. We assess the available public domain evidence on the epidemiology of SF veterinary medicines, to better understand their prevalence and distribution and their public health impact on animals and humans.

**Methods:**

Searches were conducted in Embase, PubMed, MEDLINE, Global Health, Web of Science, CAB Abstracts, Scopus, Google Scholar, Google and websites with interest in veterinary medicines quality up to 28 February 2021. Identified articles in English and French were screened for eligibility. The Medicine Quality Assessment Reporting Guidelines were used to assess the quality of prevalence surveys.

**Results:**

Three hundred and fourteen publications were included with a failure frequency (the percentage of samples that failed at least one quality test) of 6.5% (2335/35 733). The majority of samples were from post-marketing surveillance by medicines regulatory authorities of the Republic of Korea and China. A small proportion (3.5%) of samples, all anti-infectives, were from 20 prevalence surveys, with more than half (53.1%, 662/1246) collected in low-income and lower middle-income countries in Africa and Asia. The prevalence survey sample size ranged from 4 to 310 samples (median (Q1–Q3): 50 (27–80)); 55.0% of surveys used convenience outlet sampling methods. In 20 prevalence surveys more than half of the samples (52.0%, 648/1246) failed at least one quality test. The most common defects reported were out-of-specification active pharmaceutical ingredient(s) (API) content, failure of uniformity of units and disintegration tests. Almost half of samples (49.7%, 239/481) that failed API content tests contained at least one of the stated APIs below pharmacopoeial limits. Fifty-two samples (4.2% of all samples) contained one or more incorrect API. One hundred and twenty-three publications described incidents (recalls/seizures/case reports) of SF veterinary medicines in 29 countries.

**Conclusion:**

The data suggest that SF veterinary products are likely to be a serious animal and public health problem that has received limited attention. However, few studies of SF veterinary medicines are available and are geographically restricted. Lower API content and disintegration/dissolution than recommended by pharmacopoeial standards risks treatment failure, animal suffering and contribute to AMR. Our findings highlight the need of more research, with robust methodology, to better inform policy and implement measures to assure the quality of veterinary medicines within supply chains. The mechanism and impact of SF veterinary products on animal and human health, agricultural production, their economy and AMR need more transdisciplinary research.

**WHAT IS ALREADY KNOWN ON THIS TOPIC**

- Substandard and falsified (SF) veterinary medicines logically lead to negative health impacts for animals, farming communities and beyond, but data on SF veterinary medicines are scattered without global understanding of their epidemiology and impact.

**WHAT THIS STUDY ADDS**

- In the 20 studies we included aiming to understand their epidemiology, 52% of the 1246 veterinary medicine samples collected in Asia and Africa tested for quality were substandard or falsified.
- The most common reason for sample failure was out-of-specification active ingredient(s) content (46.6%, 481/1032), and 4.2% of all samples contained incorrect active ingredient(s).

**HOW THIS STUDY MIGHT AFFECT RESEARCH, PRACTICE OR POLICY**

- The results do not mean that 52% of veterinary medicines are SF globally due to limited data and issues with study methodology; more studies are needed to better inform interventions and policy.
- SF veterinary medicines may be a serious public health problem, impacting One Health, especially in low-income and middle-income countries, with little evidence on their occurrence in high-income countries.

## Introduction

Humans coexist in complex ecological relationships with other vertebrate animals, whether wild or as companions or livestock, and their environments. Omnivorous humans depend directly on healthy and productive animals for food and economic security, especially in low-income and middle-income countries where for a large proportion of people raising animals provides their livelihoods.^1^ It is estimated that 1.3 billion people raise livestock globally.^1^ Therefore, the prevention and control of animal diseases is crucial to the achievement of the Sustainable Development Goals,^1^ and constitute a global public good.^2^

Animals and humans share many of the same pathogens and three-fifths of emerging infectious human diseases between 1940 and 2004 came from animals.^2 3^ The One Health approach emphasises cross-sectoral collaboration to manage risks and consequences from the interface between human, animals and environment and improve global health security.^4^ Ensuring quality veterinary medicines is an important factor in achieving this goal. Substandard and falsified (SF) veterinary medical products will logically harm agricultural production, farming and livestock trading communities, nutrition and food security.^5 6^ The use of SF antibiotics with low active pharmaceutical ingredient (API) concentrations or low bioavailability, reflected by poor dissolution, risk subtherapeutic plasma concentration. Pathogens ‘seeing’ suboptimal concentration of antibiotics in animals may play a role in selecting for antimicrobial resistance (AMR), that can be transmitted to humans via contact with animals, food or their environment.^7^ However, the relationship between SF medicines and AMR and how it should be ranked in different communities in comparison to the other drivers of AMR has received minimal investigation.^8 9^

Pharmaceutical contamination of the environment is a complex issue with multiple sources and pathways, making it difficult to differentiate and quantify its impact.^10^ The impact of veterinary medicines on the environment depends on a number of factors including physicochemical properties, formulation, animal husbandry practices, their metabolism within the animal and environmental flows. SF veterinary medicines will add to the complexity of the issue and environmental impact of SF veterinary medicines is not discussed here.

The latest estimates of the prevalence of SF medicines for human use in low-income and middle income settings are 10.5% according to the WHO^11^ and 13.6% according to Ozawa *et al*.^12^ There are insufficient data to estimate the prevalence of SF medicines in high-income countries (HIC). Antibiotics and other anti-infective agents are the second most reported and represented class of pharmaceuticals in medicine quality surveillance and studies,^11 13 14^ and low API content and poor dissolution are the most common types of quality defects reported.^11^

In 2017, HealthforAnimals conservatively valued that the trade in global veterinary products at US$30 billion annually.^15^ Based on qualitative surveys of companies and associations, they estimated that 3% of approved veterinary products were ‘illegal’, which included falsified, substandard, unregistered/unauthorised, illegal compounded products, illegal vaccines and Intellectual Property Rights counterfeits.^15^ We aimed to explore this further, assessing the available scientific evidence on the quality of veterinary medicines, to better understand the prevalence and distribution of SF veterinary medicines and discuss their impact on animal and public health. With few new anti-infectives in the development pipeline, it is vital to understand where and how AMR originates, and a better understanding of the epidemiology of the quality of veterinary medicines would help fill in this knowledge gap.

## Methodology

### Search strategy

Reports were identified through systematic searches in PubMed, Embase, MEDLINE, Global Health, Web of Science, CAB Abstracts, Scopus in English and the first 200 results of Google and Google Scholar searches (sorted by relevance), in English and French, up to 28 February 2021. Searches were performed using veterinary medicines/drugs/products terms combined with terms relevant to medicine quality (eg, ‘falsified’, ‘substandard’) (online supplemental material 1). These key terms were adapted to each database and to search websites with interest on medicines quality or animal health (online supplemental material 2). After removal of duplicates, the titles and abstracts were first screened, and full texts of the identified articles were then assessed for eligibility. French articles identified in English searches that matched the eligibility criteria were included. Reference lists of eligible articles were manually screened for inclusion. In addition, articles identified in previous systematic reviews by our Medicine Quality Research Group (MQRG),^16 17^ but that were not captured in our searches, were included.

10.1136/bmjgh-2022-008564.supp1 Supplementary data

10.1136/bmjgh-2022-008564.supp2 Supplementary data

### Eligibility criteria

Scientific and grey literature in English or French assessing or discussing the quality of veterinary medicines were included. Studies that surveyed the quality of veterinary medicines in one or more locations (hereafter ‘prevalence surveys’) had the greatest value for this assessment. We also included articles describing the performance/development/validation of analytical techniques for the analysis of veterinary medicines, stability studies, equivalence studies, quality control studies, bioavailability studies, reports of seizures, recalls/warnings/alerts of veterinary medicines by Medicine Regulatory Authorities (MRA), pharmaceutical companies or other organisations and reports of adverse reactions where the quality of the medicine was suspected to be the cause (online supplemental material 3). We excluded articles assessing the quality of herbal medicines or dietary supplements used in animal health as pharmacopoeia reference standards and evidence base to accurately assess their quality are meagre.

10.1136/bmjgh-2022-008564.supp3 Supplementary data

### Key definitions

‘Falsified’ refers to a medical product produced with criminal intent to mislead, but without reference to intellectual property concerns implicit in the term ‘counterfeit’. ‘Substandard’ medicines, also called ‘out of specification’, are authorised medical products that ‘fail to meet either their quality standards or their specifications, or both’.^18^ As it is not possible to reliably classify a medicine as substandard or falsified without packaging analysis, products without packaging authentication that failed at least one quality test or the results are outside the acceptable limits of the chosen specifications reference (pharmacopoeia monograph or in-house specifications) are defined as ‘Substandard or Falsified’ (SorF).^16^ However, samples that contained incorrect API or no API but without packaging analysis were assumed to be falsified. Samples that passed all quality tests performed in the study with or without packaging tests are considered as good quality. Distinguishing substandard quality as a result of non-compliance with good manufacturing practice from degradation due to post-production inappropriate storage, is currently very difficult and consequently substandard medicines may also include degraded products that left the factory as good quality. We define ‘failure frequency’ (FF) as the proportion of samples that failed at least one quality test described in the report. Compounded veterinary medicines are tailored products, made by pharmacists or veterinarians for an individual animal or a small group of animals.^19^

We define ‘prevalence survey’ as a study in which medicines were collected from the pharmaceutical supply chain to assess their quality and describe the epidemiology of circulating SF medicines. Other study types are defined in online supplemental material 3. A ‘data-point’ is defined as a specific location where medicines were collected at a given time for a given study. We include co-formulated and co-blistered medicines of more than one API under the term ‘combination medicines’.

### Data analysis

Data were entered into the Medicine Quality Surveyor (https://www.iddo.org/mqsurveyor/%23veterinary) ‘Online Medicine Quality Data Manager’, an online database developed by the Infectious Diseases Data Observatory informatics team and the MQRG. Publication type and year, location where the medicines were collected (country and city, where available), sampling strategy, outlet type, stated manufacturer’s country, API/API combination name, sample size and failure frequency (with additional description of the type of failure when available), analytical/testing techniques and reference monographs/documents used.

In stability studies, only the quality data of medicines acquired prior to stress conditions were included. When the standard threshold used for the consideration of the sample as ‘pass’ or ‘fail’ was unclear, we excluded the data from the analysis.

FlySpeed SQL Query (V.3.5.4.2) was used to extract data from the online database and Microsoft Excel 2013 was used for data analysis. Qualitative variables were expressed as percentages and numbers (% (n)). Quantitative variables were expressed as median and first and third quartiles (Q1–Q3). Subgroup analyses were performed as appropriate.

This research was registered in the International Prospective Register for Systematic Review (PROSPERO, registration No: CRD42019099537) and it is reported according to the Preferred Reporting Items for Systematic Reviews and Meta-Analyses guidelines (PRISMA) (online supplemental material 4).

10.1136/bmjgh-2022-008564.supp4 Supplementary data

### Risk of bias assessment

Prevalence surveys methodology and reporting were assessed against the Medicine Quality Assessment Reporting Guidelines (MEDQUARG) and we report according to the PRISMA guidelines. MEDQUARG is a comprehensive checklist composed of 26 items proposed to be included in the reporting of medicine quality surveys.^20^ For each item, all criteria need to be fulfilled to be awarded one point. The assessment of the surveys was performed independently by two investigators and discrepancies were resolved by a third investigator. Only the prevalence surveys published as original articles in scientific journals or following the introduction/methods/results/discussion or similar style and published as reports or MSc/PhD thesis, were assessed. Articles for which assessing the prevalence of veterinary medicines quality was not the main stated aim of the study were excluded from MEDQUARG assessment.

## Results

### Literature on veterinary medicines quality

**Figure 1:**
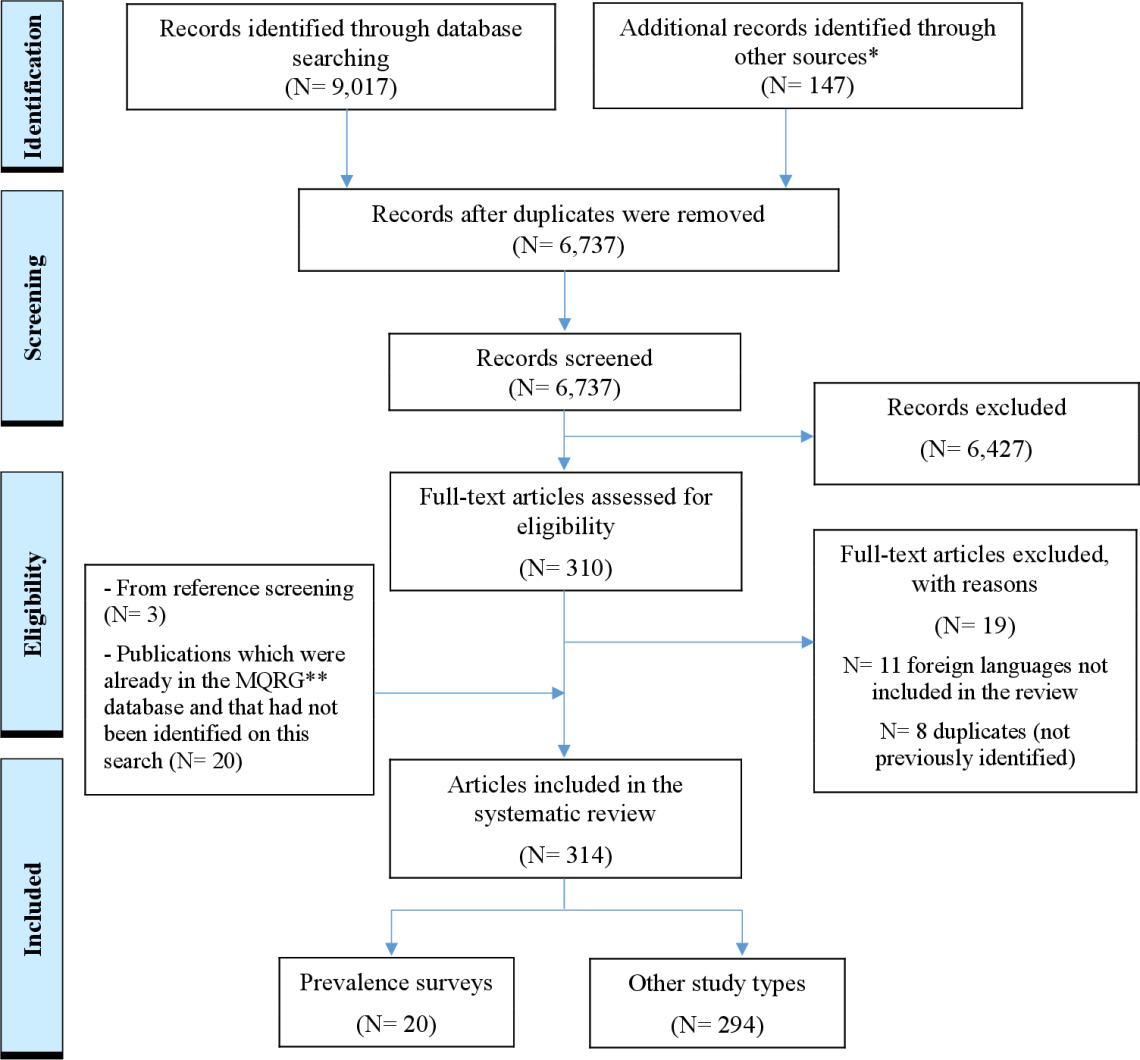
Preferred Reporting Items for Systematic Reviews and Meta-Analyses flow diagram of the selection process of the publications on veterinary medicines quality. *Websites interested in medicine quality—see (online supplemental material 2). **MQRG, Medicine Quality Research Group.

**Figure 2:**
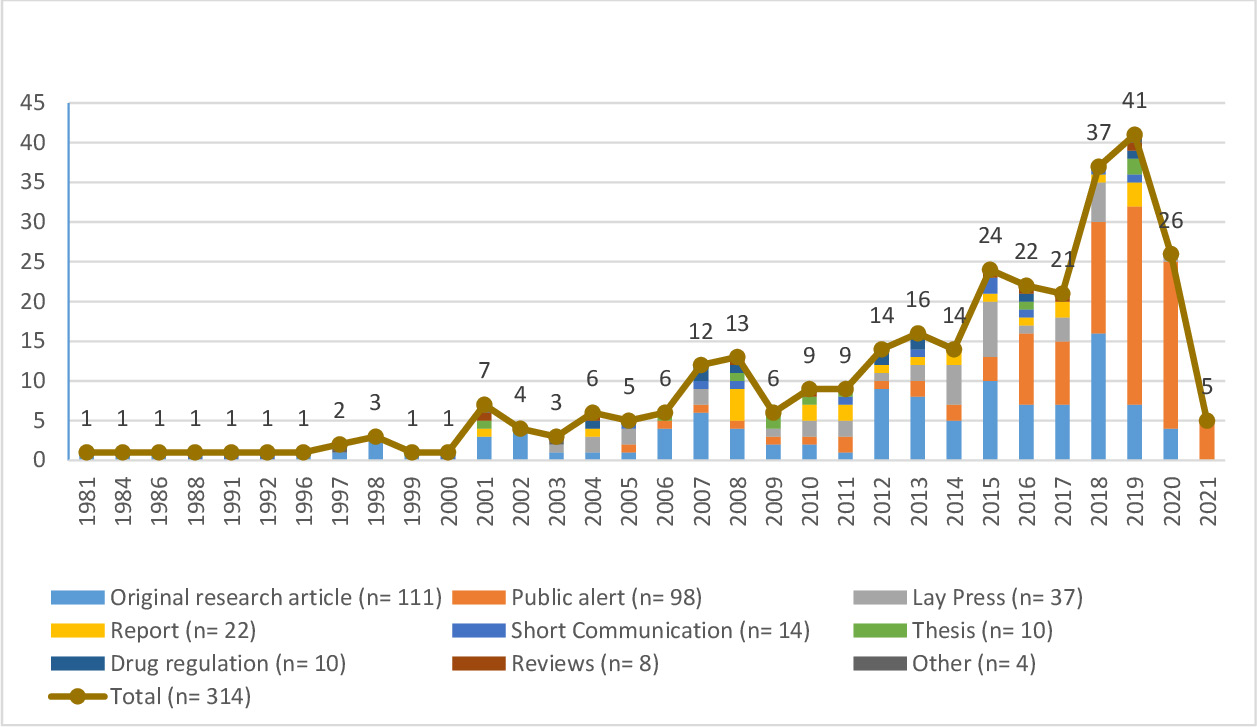
Number of publications per type and year of publication (note: publications published up to the 28 February 2021 only were included, hence the reduction in number of publications in 2021).

After duplicate removal, 6737 out of 9017 publications identified through electronic searches were screened by title and abstract (figure 1). Of these, 310 full-text publications were retrieved to assess eligibility. Nineteen were excluded. Twenty-three additional publications were found by reference screening or in the MQRG database. In total, 314 publications, published between 1981 and February 2021 were included here, of which most were original research articles (35.4%, (n=111)), public alerts by MRAs, pharmaceutical companies or other organisations (31.2%, (n=98)) and lay press articles (11.8%, (n=37)) (figure 2). Most original research articles (87.4%, (n=97)) were published in peer-reviewed journals. The number of publications per year related to veterinary medicines quality was stable between 1981 and 2000 (one to three publications per year) but has increased since 2001, reaching 41 publications in 2019, before decreasing to 26 publications in 2020.

Of the 314 publications included, 63.1% (n=198) described the quality of veterinary medicines in specific locations and times with a total of 543 data points. Of these, 12.1% (n=24) were analytical technique development/validation studies, 10.1% (n=20) prevalence surveys, 8.1% (n=16) routine quality control studies, 5.6% (n=11) equivalence studies, 2 (1.0%) stability studies and 2 (1.0%) were other publication types (online supplemental material 5). Others were recall/warning/alert (36.9%, n=73), seizures (24.2%, n=48) and case reports (1.0%, n=2).

10.1136/bmjgh-2022-008564.supp5 Supplementary data

Most publications without data point information were discussions of different aspects of the quality of veterinary medicines (38.0%, n=42) and analytical technique development/validation (33.0%, n=40) without sufficient information on the samples used.

A total of 35 733 samples of 152 different APIs or combinations of APIs and of 14 different veterinary vaccines were collected in 38 countries and tested for quality, mainly in routine MRA quality control analysis (n=34 202, 95.7%). Most of these samples were described in one article reporting 10 years (2006–2016) of post-market surveillance in the Republic of Korea (n=18 213),^21^ and one report of post-market surveillance in China in 2012 (n=14 373).^22^ Prevalence surveys included 1246 samples (3.5%), equivalence studies 188 (0.5%), analytical technique development/validation studies 79 (0.2%) and 14 samples were tested in stability studies. Four additional samples from a case report were also included.^23^ Of all samples, 6.5% (n=2335) failed at least one quality test. Of the failing samples 93.7% (n=2187) were classified as SorF because no packaging analysis to assess the authenticity of the samples had been performed, 3.7% (n=87) falsified and 2.6% (n=61) substandard. All reports were of anti-infectives, including three reports of SF veterinary vaccines^21 22 24^ with no reports of SF medicines for non-communicable diseases found.

All data are mapped in the Infectious Diseases Data Observatory Medicine Quality Surveyor system (https://www.iddo.org/mqsurveyor/%23veterinary) and can be downloaded.

### Prevalence surveys

Twenty prevalence surveys, published between 2001 and 2020, were included (online supplemental material 6). Overall, 1246 samples of 31 different APIs or combinations of APIs were collected in 24 countries with 173 data points. The sample size ranged from 4 to 310 samples per survey with a median (Q1–Q3) of 50 (27–80) samples per survey.

10.1136/bmjgh-2022-008564.supp6 Supplementary data

The overall FF in prevalence surveys was 52.0% (648/1246). Eleven (55.0%) prevalence surveys used convenience sampling to select outlets to be sampled, three (15.0%) used random sampling, one (5.0%) used a combination of random and convenience sampling and for five surveys (25.0%) the sampling methodology was not given or clearly stated. Samples obtained by random selection had the highest FF (62.2%, 171/275), followed by those obtained through convenience sampling (FF=61.4% 231/376) and through unstated/unclear sampling strategy (FF=41.3%, 246/595).

Reference threshold to classify the samples as pass or fail of ±10% compared with the declared label claim was chosen arbitrarily (without reference to a pharmacopoeia) in seven surveys. The European Pharmacopoeia, the United States Pharmacopeia, the British Pharmacopoeia and the Veterinary Pharmacopoeia of the People’s Republic of China were used for reference ranges for six, four, four and one studies, respectively (online supplemental material 6). One survey in African countries used a monograph of the World Organisation for Animal Health,^25^ and one survey used the International Pharmacopoeia.^26^

More than half (53.1%, n=662) of samples were collected from low-income countries (LIC), mainly in Africa, followed by 37.6% (n=468) from upper middle-income countries, 6.1% (n=76) from lower middle-income countries (LMIC) and 2.5% (n=31) from high-income settings (table 1). Five samples were obtained through the internet (country where samples were shipped from was not specified). The country of collection of four samples was not stated.

**Table 1:** Veterinary medicine failure frequency by continent/country in prevalence surveys^63^

Continent	Income level	Country	No. data points	Failure frequency % (n/N)
Asia				**72.1 (165/229)**
	LMIC	Viet Nam	47	77.3 (92/119)
	UMIC	China	7	76.1 (54/71)
	LMIC	Pakistan	1	50.0 (4/8)
	High-income setting	Hong Kong, SAR, China	4	48.4 (15/31)
Africa				**47.3 (479/1012)**
	LMIC	Cameroon	10	84.0 (63/75)
	LIC	DR Congo	1	66.7 (2/3)
	LMIC	Senegal	17	65.2 (58/89)
	LIC	Rwanda	6	65.1 (54/83)
	LIC	CAR	1	60.0 (3/5)
	LIC	Madagascar	4	57.9 (33/57)
	LIC	Benin	6	53.4 (39/73)
	LMIC	Ghana	5	52.0 (13/25)
	LMIC	Angola	1	50.0 (1/2)
	LIC	Niger	4	48.8 (21/43)
	LIC	Chad	1	46.7 (7/15)
	LMIC	Côte d'Ivoire	14	45.4 (64/141)
	LMIC	Nigeria	5	44.4 (4/9)
	LIC	Togo	14	42.9 (33/77)
	LIC	Burkina Faso	3	31.5 (17/54)
	LIC	Ethiopia	4	28.0 (14/50)
	LIC	Mali	13	26.4 (52/197)
	UMIC	Namibia	1	0.0 (0/5)
	LIC	Malawi	1	0.0 (0/3)
	LIC	Mozambique	1	0.0 (0/2)
	Unknown*	Unknown*	1	25.0 (1/4)
Internet	Not applicable	Unknown*	1	80.0 (4/5)
Total			**173**	**52.0 (648/1246)**

*Country where samples were collected/shipped to was not reported.

CAR, Central African Republic; DR Congo, Democratic Republic of the Congo; Hong Kong SAR, Hong Kong Special Administrative Region; LIC, low-income country; LMIC, lower middle-income country; UMIC, upper middle-income country.

All samples with known location of collection were procured in Africa (81.2%, n=1012) and Asia (18.4%, n=229). The failure frequency in Asia was the highest (72.1%, 165/229), with samples collected in Vietnam, China, Pakistan and Hong Kong Special Administrative Region of China (FF of 77.3% (92/119), 76.4% (54/71), 50.0% (4/8) and 48.4% (15/31), respectively). The FF in Africa was 47.3% (479/1,012) and the highest FF was found in Cameroon (84.0%, 63/75). The country with the highest number of samples collected was Mali (n=197) with a FF of 26.4%.

The anti-infective diminazen was the most frequently collected API in prevalence surveys, with 310 samples included (24.9%), followed by oxytetracycline (21.9%, n=273) and combinations of diminazen-phenazone (12.8%, n=155) with FFs of 45.5%, 41.4% and 63.2%, respectively (table 2).

**Table 2:** Veterinary medicine failure frequency by API/API combinations in prevalence surveys

Medicine class/subclass	API/API combination	No. data points	Failure frequency % (n/N)
Antibiotics		88	55.3 (271/490)
Antibiotics (single API formulations)		53	49.1 (192/391)
	Amoxicillin	2	100.0 (16/16)
	Procaine benzylpenicillin (penicillin G)	2	100.0 (7/7)
	Sulfamonomethoxine	1	100.0 (4/4)
	Neomycin	1	100.0 (1/1)
	Ciprofloxacin	2	83.3 (5/6)
	Doxycycline	3	80.0 (4/5)
	Florfenicol	8	58.3 (28/48)
	Enrofloxacin	3	46.4 (13/28)
	Oxytetracycline*	30	41.4 (113/273)
	Tilmicosin	1	33.3 (1/3)
Combination of antibiotics		35	79.8 (79/99)
	Doxycycline–oxytetracycline†	1	100.0 (4/4)
	Amoxicillin–colistin	1	100.0 (3/3)
	Amoxicillin–tylosin	1	100.0 (3/3)
	Enrofloxacin–oxytetracycline†	1	100.0 (3/3)
	Trimethoprim–colistin	1	100.0 (3/3)
	Amoxicillin–ciprofloxacin	1	100.0 (2/2)
	Doxycycline–florfenicol	2	100.0 (2/2)
	Erythromycin–sulfamethoxazole	1	100.0 (2/2)
	Oxytetracycline–sulfadimethoxin–ormetoprim	1	100.0 (2/2)
	Sulfamethoxazole–trimethoprim–rifampicin	1	100.0 (2/2)
	Doxycycline–rifampicin†	1	100.0 (1/1)
	Enrofloxacin–ciprofloxacin–amoxicillin†	1	100.0 (1/1)
	Enrofloxacin–oxytetracycline–florfenicol†	1	100.0 (1/1)
	Erythromycin–rifampicin†	1	100.0 (1/1)
	Florfenicol–amoxicillin	1	100.0 (1/1)
	Florfenicol–cefalexin	1	100.0 (1/1)
	Streptomycin–neomycin†	1	100.0 (1/1)
	Sulfadimethoxin–ormetoprim	1	100.0 (1/1)
	Sulfadiazine–trimethoprim	4	92.3 (12/13)
	Oxytetracycline–colistin	1	83.3 (10/12)
	Tylosin–gentamicin	1	83.3 (5/6)
	Sulfamethoxazole–trimethoprim	4	75.0 (6/8)
	Doxycycline–tylosin	2	71.4 (10/14)
	Neomycin–colistin	1	33.3 (1/3)
	Thiamphenicol–sulfamethoxazole	1	33.3 (1/3)
	Gentamicin–colistin	1	0.0 (0/3)
	Oxytetracycline–streptomycin	1	0.0 (0/3)
Antiparasitics		85	49.9 (377/756)
Endectocides		9	55.1 (27/49)
	Ivermectin	9	55.1 (27/49)
Antiprotozoals		31	45.7 (176/385)
	Isometamidium	18	46.7 (35/75)
	Diminazen	13	45.5 (141/310)
Antihelmintics		23	45.5 (76/167)
	Closantel	1	100.0 (3/3)
	Levamisole	4	81.3 (26/32)
	Praziquantel	1	40.0 (2/5)
	Albendazole	17	35.4 (45/127)
Combination of antiprotozoals and analgesics–antipyretics		22	63.2 (98/155)
	Diminazen–phenazone	22	63.2 (98/155)
Total		173	52.0 (648/1246)

Combination medicine includes both co-formulated and co-blistered APIs.

*One sample of oxytetracycline was stated to also contain sulfafurazole but it is unclear in the publication whether it was tested in the laboratory or not.

†These medicines were stated to also contain berberine/allicin/beta-glucan/lamivudine but it is unclear in the publications whether they were tested in the laboratory or not.

API, active pharmaceutical ingredient.

The highest FF was for antibiotics (55.3%, 271/490), followed by antiparasitics (49.9%, 377/756). Combinations of antibiotics or of antiparasitics had the highest FF ((79.8%, (79/99) and 63.2% (98/155), respectively). Most samples were tested by more than one quality test (75.9%, n=946). Almost half of the samples tested for API content failed the tests (46.6%, 481/1032), 29.3% (54/181) failed uniformity of unit tests, 23.1% (31/134) failed disintegration tests and 1.1% (8/718) failed tests for packaging/label/physical appearance inspection (online supplemental material 7). No samples failed impurity/contaminant/related substances nor sterility tests out of the 27 and 120 tests performed, respectively. No samples were tested for dissolution in prevalence surveys.

10.1136/bmjgh-2022-008564.supp7 Supplementary data

Of 481 samples that failed API content assays, 41 samples (8.5%) contained none of the stated API(s). Of those, 19 samples (46.3%) contained incorrect API(s) (online supplemental material 8). Almost half (49.7%, n=239) contained lower than the pharmacopoeial recommended percentage of at least one of the APIs, with percentages ranging from 0.001% (one enrofloxacin sample in a combination formulation) to 89.9%. Twenty-two per cent (n=106) of samples contained higher amounts of API than pharmacopoeial limits, with the highest %API of 284.0% for florfenicol in a combination formulation. Twenty-one samples (4.4%) stated to contain several APIs actually contained a mixture of low/high %APIs and/or no API (eg, low % of API-A and high %API-B or no API-B). Thirty-three samples (6.9%) contained variable amounts of the stated API(s) and an incorrect API. For 41 samples (8.5%) there was insufficient information to determine whether they contained higher or lower %API than pharmacopoeial limits.

10.1136/bmjgh-2022-008564.supp8 Supplementary data

Among the 52 samples containing incorrect API(s) (5.0% of the samples tested for API content; 4.2% of all the samples tested in prevalence surveys), amiodarone was identified in six samples stated to contain penicillin G. Other incorrect APIs detected were antibiotics used in other veterinary and/or human medicines: enrofloxacin, sulfadimidine, sulfamonomethoxine, amoxicillin, sulfadiazine, sulfamethazine, sulfamethoxazole, florfenicol and cephalexin.

Most of the samples (85.5%, 882/1032) tested for API content were analysed by HPLC (High Performance Liquid Chromatography) or a derivative technique (coupled with various detectors), and 124 (12.0%) by UV(Ultraviolet)-visible spectrophotometry. For 26 samples (2.5%), no details on the technique used were given.

Besides a 100% FF for one sample collected directly from a manufacturer that failed the quality tests,^7^ the highest FF was for samples collected in ‘chemical shops’ (80.2%, 105/131), samples obtained from online pharmacies (80.0%, 4/5) and farms (75.0%, 3/4) (online supplemental material 9). The FF for samples collected in veterinary clinics/hospitals/health centres was 60.0% (18/30), in veterinary medicines outlets 58.9% (43/73), in unlicenced/unregistered outlets 45.9% (90/196) and samples collected in wholesalers/distributors facilities was 43.8% (56/128). A large number of samples (n=523) were collected from a combination of outlets including private and government facilities without breakdown of the FF by outlet type, with an overall FF of 57.0% (298/523). There were no details on where 155 samples were collected from, with an FF of 19.4%.

10.1136/bmjgh-2022-008564.supp9 Supplementary data

The highest failure frequency was for samples stated as made by American manufacturers (FF=77.8%, 14/18), followed by Asian (FF=76.0%, 139/183), African (FF=66.7%, 4/6) and European manufacturers (64.4%, 67/104) (online supplemental material 10). Manufacturer’s origin was not specified for 935 samples (FF=45.3%, 424/935).

10.1136/bmjgh-2022-008564.supp10 Supplementary data

### Quality of studies assessment

**Figure 3:**
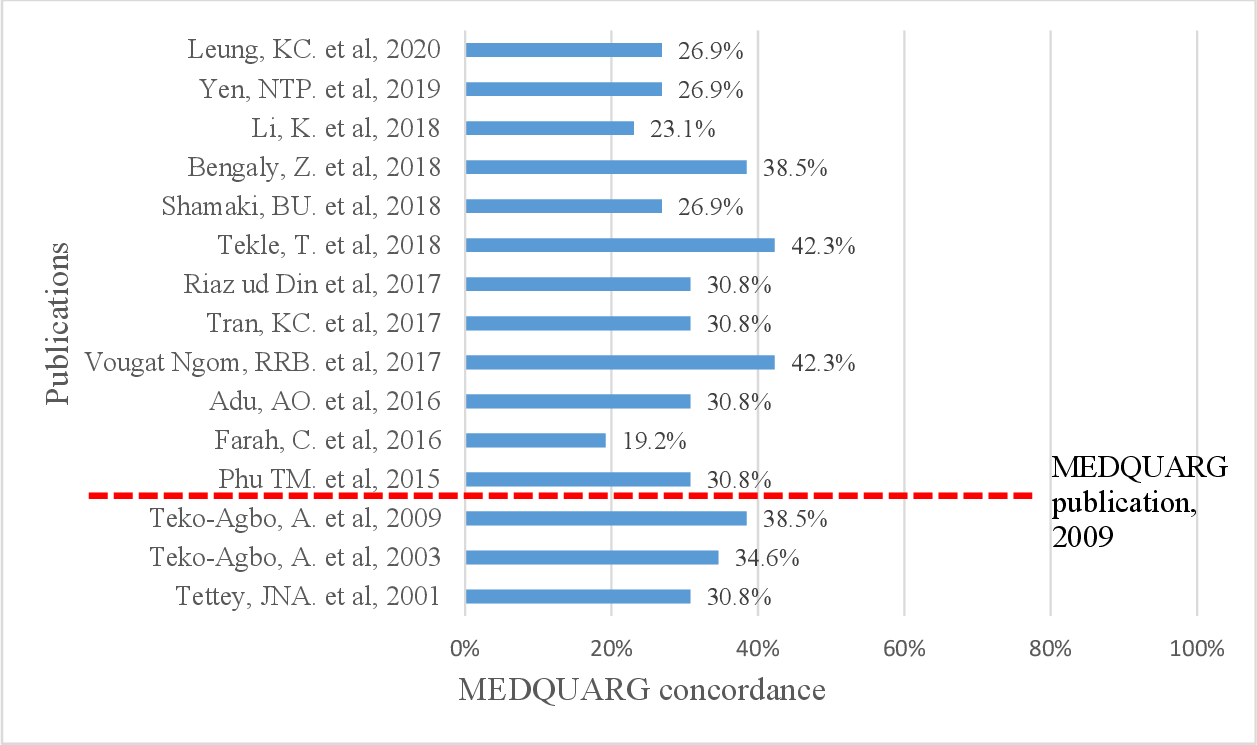
Percentage of concordance of the 15 prevalence surveys with the 26 items included in MEDQUARG checklist. MEDQUARG, Medicine Quality Assessment Reporting Guidelines.

Fifteen prevalence surveys on the quality of veterinary medicines met the inclusion for appraisal on reporting quality using MEDQUARG. The median percentage of concordance to MEDQUARG criteria was 30.8% (26.9%–36.5%) (figure 3).

Twelve prevalence surveys were reported after the publication of the MEDQUARG in 2009, but none of these stated the use of these guidelines for reporting the findings. Two (13.3%) studies provided definitions on the quality of medicines or used the WHO definitions (online supplemental material 11). One (6.7%) study reported how the sample collectors carried out the sampling and in what guise. No study gave all the sampling design information listed in MEDQUARG Item 6. Only one study stated to have shared the findings with the appropriate MRA or had a representative of the country MRA as an author of the article.

10.1136/bmjgh-2022-008564.supp11 Supplementary data

### Equivalence studies

We found 11 equivalence studies with 28 data points, including 188 samples with a median of 10 (8–13) samples collected per study. The studies included samples of 10 different APIs/API combinations collected in six countries (online supplemental materials 12 and 13). In total, 101 out of 188 (53.7%) samples failed at least one quality test. Of all the samples tested, 45.7% (n=86) were SorF and 8.0% (n=15) were substandard. Most samples in equivalence studies were compounded medicines (66.5%, 125/188) with an FF of 50.4% (63/125). One generic medicine out of eight (12.5%) failed at least one test. For other samples (37/55) it was not clearly stated whether the samples were generics or innovators. The FF due to uniformity of units failure was the highest (57.9%, 11/19) followed by API content failure with FF of 43.1% (81/188), and 23.1% (25/108) samples failed dissolution test.

10.1136/bmjgh-2022-008564.supp12 Supplementary data

10.1136/bmjgh-2022-008564.supp13 Supplementary data

### Seizures, recalls and case reports

One hundred and twenty-three publications describing recalls/warning/alerts (n=73), seizures (n=48) and case reports (n=2) of SF veterinary medicines in 29 countries were found (online supplemental material 14). A presentation provided a list of 31 SF veterinary products found in 18 countries and reported to the WHO Global Surveillance and Monitoring System between 2017 and 2019, but without details of the product defects.^27^

10.1136/bmjgh-2022-008564.supp14 Supplementary data

Among the publications on seizures, 19 were of unauthorised products, 9 of products containing out-of-specifications API content, 6 described illegal production of veterinary medicines and 14 described the seizure of veterinary products with no information on the quality defects. Two reports were of illegal production, involved bulk oxytocin that was smuggled and formulated as oxytocin injections by unlicenced Chinese manufacturers.^28^ The injections were manufactured ‘without blister pack’ and ‘with an intention to divert the stocks for veterinary use’.

## Discussion

We synthesised data on SF veterinary medicines from different resources, available in the public domain, from 1981 to February 2021. The overall FF was 6.5% from a total of 35 733 samples. With the paucity of data and their quality, this does not mean that 6.5% of veterinary medicines are SF products. A large proportion of samples were from routine MRA quality control analysis in the Republic of Korea and China, and only 3.5% were from the few prevalence surveys. Of the 1246 samples from 20 studies designed to assess SF prevalence, 52.0% failed at least one quality test. These prevalence surveys focused on the quality of antimicrobials and samples were collected in Africa and Asia. Lower API content than stated was the most common reason for failure and 52 samples contained incorrect unstated APIs of variable amounts. The lower API content could be due to poor manufacturing practice or degradation during storage but products with very low, no API or incorrect API content are likely to be due to fraud. No reports on the quality of veterinary anaesthetics or medicines for non-communicable diseases were found, but SF veterinary vaccines have been reported in post-marketing surveillance.^21 22 24^ There have also been lay press reports, for example, from South Africa.^29^

Antibiotics are the most commonly used type of veterinary medicines,^9^ and it is therefore unsurprising that all samples from prevalence surveys contained at least one antimicrobial. In China, the use of antibiotics in animal production accounts for more than half of the national antibiotics marketed for use in animals and humans, mostly being used in pig and broiler farming.^30^ The commonly used antibiotics in food animal production are tetracyclines, penicillins, macrolides and sulphonamides.^30 31^ All these classes of antibiotics were included in the prevalence surveys and the highest proportion of samples collected and tested for quality was for oxytetracycline for which two-fifth of the samples tested failed at least one test. The FF for antibiotics was higher for combination of APIs than single API formulations, perhaps because of the greater complexity of manufacture. Diminazen as a single agent and in combination had the largest sample size of all antiparasitic agents tested, with a high FF (45.5%).

In prevalence surveys, samples failed mainly due to out of specification API(s) content, uniformity of dosage units and disintegration. This is similar to observations for antidiabetics and cardiovascular medicines for use in humans.^17 32^ In the equivalence studies, almost one-fourth of the samples tested for dissolution failed, but dissolution test was not performed on any samples in the prevalence surveys despite its importance as a proxy for bioavailability.^32 33^ The high dissolution failure rate in equivalence studies highlights that prevalence surveys that neglect dissolution testing may fail to detect poor quality samples, underestimating the prevalence of SF medicines. Products with low API content or low bioavailability, due to poor disintegration or dissolution, can lead to treatment failure, potentially resulting in unnecessary use of more expensive secondary treatments, animal suffering and economic loss to farmers. SF medicines were thought to be a contributing factor to treatment failures reported by farmers in Ethiopia and Cameroon.^34 35^

Antimicrobials with below API content specifications will likely contain insufficient API content to kill the pathogen, yet enough to engender the development and spread of AMR.^9^ Likewise, SF combination antibiotics risk both treatment failure and the development of AMR for one or all APIs as desired synergistic and protective effects may not be achieved. Transmission of resistant zoonotic pathogens to humans can directly cause untreatable or resistant infections in livestock workers in addition to the wider impact on human health.^30 36–39^ For example, the extensive use of avoparcin in agriculture and fluoroquinolone in chicken production may have contributed to the emergence of vancomycin resistance and fluoroquinolone-resistant campylobacter in humans.^40^ Among food animals in which mass medication with antibiotics is often used for growth promotion and for metaphylaxis and prophylaxis, products containing higher API than the stated amount could directly harm animals and humans as a result of overdosage. Furthermore, the resulting high level of residue in the environment and food chain can contribute to AMR.^9 30 31 40^

Fifty-two samples (4.2% of all samples tested in prevalence surveys) contained undeclared API, ranging from negligible to 55.8% (w/w) of the unit content, with or without the presence of the labelled API(s). This may be due to a gross negligence or fraudulent intent during production. The cross contamination of ingredients used in human and animal medicines is possible with poor manufacturing practices when produced in the same factory.^41^ Most undeclared APIs identified in the prevalence survey samples were broad spectrum antibiotics used in humans and/or animals, with the exception of amiodarone (of unknown quantity) identified in six injectable long-acting benzylpenicillin samples from Cameroon.^42^ Amiodarone is a cardiac medicine commonly used in humans and off licence in animals to treat arrhythmias. The implication of such cryptic exposure to amiodarone is unknown but accumulation is possible due to its long elimination half life. At a significant quantity or in prolonged exposure, amiodarone can disrupt thyroid and liver function.

Most samples in prevalence surveys were collected in African countries and SF veterinary medicines were identified in 17 African countries with an overall FF of 47.3%. In Asia, SF were identified in four countries, with a failure frequency of 72.1%. The highest FF were found in Cameroon, Vietnam and China. A lack of or weak veterinary medicine regulatory capacity, poor access to registered outlets, unaffordable authentic products and complex manufacturing and unregulated trading are potential drivers.^15 43^ In many countries, veterinary services and product regulatory systems are weakly structured, with fewer qualified personnel for registration and sale of veterinary medicines and vaccines in comparison to systems for human medicines.^44^ A regional registration process for veterinary products was established in West Africa under the ‘Union Economique et Monétaire Ouest Africaine’ in 2006 to provide control on importation and sales. However, only 20% of veterinary products submitted to the system were processed in the subsequent 10 years because of authorisation delays.^44^ In most Eastern and Southern African countries, pharmacy boards (or equivalent) regulate the registration, distribution and use of veterinary medicines but they have been stated to lack financial and human resource capacity to perform effectively,^45^ leading to unrestricted importation and distribution of veterinary medicines.^44^

We found only one prevalence survey, with very limited sample size, in China, and none in India, although China and India are reported as the main suppliers of veterinary medicines globally.^15^ No prevalence surveys were identified in European, American, Australasian or Pacific countries. We found survey data from only one high-income setting, Hong Kong Special Administrative Region (SAR), China. The under representation of samples from high-income settings is similar to prevalence studies of human medicines^11 12 16 46^ and may be a result of strong supply chain regulations and/or higher confidence on supply chains.^47^ However, the high FF found in Hong Kong SAR (although only 31 samples were tested) and high FF of human cardiovascular medicines found in high-income settings indicate that these settings are not immune from the reach of SF medicines.^46^ Likewise, post-marketing assessment of pharmaceutical products sampled in the European Union over 20 years by the European Medicines Agency identified that 9% of veterinary medicines and 25% of veterinary vaccines failed laboratory tests or had regulatory issues.^24^

Only one survey was found on the quality of veterinary medicines acquired online, with a small number of samples tested, rendering interpretation difficult. This data paucity is surprising, especially considering the alarmingly high proportion of veterinary antibiotics available online without prescriptions.^48^ Furthermore, the ease of international parcel shipments have increased internet sales of veterinary medicines for companion animals in some parts of the world.^49^ The WHO suggests that half of human medicines obtained through the internet may be falsified, though information on the e-commerce market size and proportion of SF veterinary medicines being traded through the internet are lacking.^50^ The trading of SF veterinary medicines will likely increase as the pharmaceutical e-commerce market continues to grow, increasing interest in post-market surveillance globally.^15 51^ For example, publications by Health for Animals^52^ and the World Organisation for Animal Health^53 54^ propose SF reporting systems analogous to that of the WHO for human SF medicines (https://www.who.int/who-global-surveillance-and-monitoring-system).

### Limitations

Limitations include that searches were conducted only in English and French, and we identified recalls/seizures/case reports mainly from searches in a limited number of MRA’s websites and other websites interested in medicine quality. Routine MRA post-marketing surveillance provides valuable and actionable data that is specific to the region to guide appropriate actions but with frequently undisclosed sampling and analysis techniques, many prevalence studies continue to provide key information in the public domain. Furthermore, unpublished post-marketing surveillance results from other MRAs and the pharmaceutical industry could not be captured. Spanish and Portuguese were not included in our searches, risking the exclusion of articles, especially in Latin America.

Most studies were of small sample size and used convenient sampling which risk bias. The quality of reporting of prevalence surveys was also poor as reflected by the low MEDQUARG scores. None of the surveys completed after the publication of the MEDQUARG in 2009 followed them to report the results, although these guidelines focused on human medicines.

In most prevalence surveys, we found limited information on samples and/or samples quality classified by outlet type sampled (eg, licenced vs unregistered outlet) or stated country of manufacture. We thus did not perform further analysis that, although important to better inform policy, could lead to misleading results and interpretation.

The quality of samples was assessed by different pharmacopoeia references as indicated in the publications and no independent confirmation tests were performed.

### Recommendations

Circulation and use of SF antimicrobials in animals will impact on animal health, economic loss to farmers and agricultural traders, safety of animal food products for human and potential emergence and spread of AMR. There are at least three major impacts of SF veterinary medicines containing high, low, no or incorrect API content, or poorly dissolving, on human health. First, their impact on health, productivity and well-being of domestic, companion and wild animals, with implications for food security and economy. Second, human health impacts when humans inappropriately use veterinary medicines.^55^ Third, SF antimicrobials used in animals will impact, in currently poorly understood ways, the epidemiology of AMR in human and animal pathogens, with multiple knock-on consequences for One Health.

How SF antimicrobials may drive AMR has received scant attention,^56–58^ and none that we can find on the evolution and spread of AMR due to SF veterinary medicines. Public health implications on AMR of SF veterinary antimicrobials will depend on the pharmacokinetic–pharmacodynamic mechanisms of the relationship between antimicrobial concentrations and pathogens in different animal and human body compartments. The risk benefit of interventions to reduce the prevalence of SF veterinary antimicrobials when used as promoters versus those used for treatment, for example, will differ with global calls to ban the use of antimicrobials as growth promoters.^59^ Veterinary medicines used in food producing animals and excreted into the environment will circulate to humans and other vertebrates, domestic and wild. As for zoonotic diseases, the separation between medicines for livestock, wild vertebrates and pets and those for humans is artificial and there needs to be further transdisciplinary research to understand the One Health ecology of use, especially for antimicrobials and their comparative role in engendering AMR.

People have been recorded as using veterinary antimicrobials, such as the widespread unapproved use of ivermectin for COVID-19,^60^ and criminals have used veterinary medicines such as levamisole as adulterants in falsified human medicines and cocaine.^61^ The widespread global increase in pet ownership over the last 70 years as companions to humans,^62^ and the financial implications of costs of care and medicines suggests that SF medicines will also have an impact on the economy and well-being of families with pets.

A lack or weak medicines regulatory capacity, poor access to registered outlets and cost of medical products along with complex manufacturing and trading are some of many potential drivers of SF medicines for both humans and animals. Multidisciplinary approaches involving among others, politicians, police, customs, medicine regulatory authorities, scientists, pharmaceutical companies, veterinarians, farmers and the public are needed to address the issue.

In view of the limitations described above, prevalence surveys with robust methodology and larger sample sizes, in wider geographical regions including LIC and HIC and e-commerce marketplaces are needed for a more comprehensive epidemiological information on the quality of veterinary medicines. All prevalence surveys should be reported to the MRA to ensure appropriate actions are taken to address specific issues identified in the region.

## Conclusion

These results highlight that there are few data on the quality of veterinary medicines in many parts of the world, including high-income settings and online marketplaces. Nonetheless the proportion of samples failing quality tests is alarming. For a better insight of this global issue future studies need to fill knowledge gaps with robust surveys with adapted sampling methodology to estimate the prevalence of SF veterinary medicines, examination of trends over time and their impact on animals, humans and their economy. More global investment is needed in the enforcement of regulations on pharmaceutical supply chain and quality control system for veterinary medicines.

## Data Availability

All data relevant to the study are included in the article or uploaded as supplementary information.
