# Supplementary material for: The quality of veterinary medicines and their implications for One Health
Source: BMJ Glob Health. 2022 Aug 1;7(8):e008564. doi: 10.1136/bmjgh-2022-008564 (PMC9351321; doi:10.1136/bmjgh-2022-008564)
Supplement: Supplementary data [file bmjgh-2022-008564supp001.pdf]

The quality of veterinary medicines and their implications for One Health

Supplemental material 1. Search terms used for the systematic review of the quality of veterinary medicines

**Sources**	**English search terms**
Pubmed	(((((("Veterinary Drugs"[Mesh]) OR "Veterinary Medicine"[Mesh])) OR veterinary)) AND ((((((((((("Counterfeit Drugs"[Mesh]) OR counterfeit*) OR fake*) OR spurious) OR substandard) OR falsified) OR drug quality) OR medicine* quality) OR pharmaceutic* quality) OR false* label*) OR poor quality drug*)) OR (((veterinary)) AND ((((((((((("Counterfeit Drugs"[Mesh]) OR counterfeit) OR fake) OR spurious) OR substandard) OR falsified) OR "drug quality") OR "medicine* quality") OR "pharmaceutic* quality") OR false* label*) OR "poor quality drug*"))
Medline	(veterinary drugs or veterinary medicine or veterinary.ti,ab.) and (counterfeit drugs or counterfeit* or fake* or spurious or substandard or falsified or (drug adj2 quality) or (medicine adj2 quality) or (pharmaceutic* adj2 quality) or "false* label*" or "poor quality drug*" or (quality adj2 control*) or (quality adj2 assess*)).ti, ab.
Web of science	("Veterinary Drugs" OR "Veterinary Medicine" OR veterinary) AND ("Counterfeit Drugs" OR counterfeit* OR fake* OR spurious OR substandard OR falsified OR drug quality OR medicine* quality OR pharmaceutic* quality OR false* label* OR poor quality drug*)
CAB Abstract	(veterinary drugs or veterinary medicine or veterinary.ti,ab.) and (counterfeit* or fake* or spurious or substandard or falsified or (drug adj2 quality) or (medicine adj2 quality) or (pharmaceutic* adj2 quality) or "false* label*" or "poor quality drug*" or (quality adj2 control*) or (quality adj2 assess*)).ti, ab.
Embase	("Veterinary Drugs" OR "Veterinary Medicine" OR veterinary) AND ("Counterfeit Drugs" OR counterfeit* OR fake* OR spurious OR substandard OR falsified OR drug quality OR medicine* quality OR pharmaceutic* quality OR false* label* OR poor quality drug*)
Global Health	((veterinary products or veterinary medicine).mp. or veterinary.ti,ab.) and (counterfeit* or fake* or spurious or substandard or falsified or (drug* adj2 quality) or (medicine* adj2 quality) or (pharmaceutic* adj2 quality) or "false* label*" or "poor quality drug*" or (quality adj2 control*) or (quality adj2 assess*)).ti,ab.
Scopus	TITLE (veterinary) OR ABS (veterinary) AND TITLE (counterfeit* OR fake* OR spurious OR substandard OR falsified OR (drug* W/2 quality) OR (medicine* W/2 quality) OR (pharmaceutic* W/2 quality) OR "false* label*" OR "poor quality drug*" OR (quality W/2 control*) OR (quality W/2 assess*)) OR ABS (counterfeit* OR fake* OR spurious OR substandard OR falsified OR (drug* W/2 quality) OR (medicine* W/2 quality) OR (pharmaceutic* W/2 quality) OR "false* label*" OR "poor quality drug*" OR (quality W/2 control*) OR (quality W/2 assess*))
Google	(veterinary) (counterfeit*|fake*|spurious|substandard|falsified|"drug quality"|"medicine quality"|"pharmaceutic* quality"|"false* label*"|"poor quality drug*"|"quality control*"|"quality assess*")

The quality of veterinary medicines and their implications for One Health

Google Scholar	(veterinary) (counterfeit*|fake*|spurious|substandard|falsified|"drug quality"|"medicine quality"|"pharmaceutic* quality"|"false* label*"|"poor quality drug*"|"quality control*"|"quality assess*")
**Sources**	**French search terms**
Google	(“médicament vétérinaire”|vétérinaire) (contrefaçon|"faux médicament"|"médicament fallacieux"|falsifié|sous-standard|“qualité pharmaceutique”|“qualité des médicaments”)
Google Scholar	(“médicament vétérinaire”|vétérinaire) (contrefaçon|"faux médicament"|"médicament fallacieux"|falsifié|sous-standard|“qualité pharmaceutique”|“qualité des médicaments”)
