# Supplementary material for: The quality of veterinary medicines and their implications for One Health
Source: BMJ Glob Health. 2022 Aug 1;7(8):e008564. doi: 10.1136/bmjgh-2022-008564 (PMC9351321; doi:10.1136/bmjgh-2022-008564)
Supplement: Supplementary data [file bmjgh-2022-008564supp002.pdf]

The quality of veterinary medicines and their implications for One Health

Supplemental material 2. Websites used for information searching about substandard and falsified veterinary medicines

	**Website names and hyperlinks**
International organizations and NGOs	European Alliance for Access to Safe Medicines (EAASM)
	Fondation Chirac - Agir au service de la paix
	International Criminal Police Organization (INTERPOL)
	United Nations Office on Drugs and Crime (UNODC)
	Médecins Sans Frontières Access Campaign
	The partnership for SAFEMEDICINES
	Third World Network
	United States Pharmacopeia Promoting Quality of Medicines (USP-PQM)
	World Organization for Animal Health (OIE)
	Research Institute on Contemporary Southeast Asia (IRASEC)
	The Anti-Counterfeit Group (ACG)
	Global Anti-Counterfeiting Group (GACG Network)
	International AntiCounterfeiting Coalition (IACC)
	WHO Essential Medicines and Pharmaceutical Policies
	Institute of Research Against Counterfeit Medicines (IRACM)
	Permanent Forum on International Pharmaceutical Crime (PFIPC)
	Pharmaceutical System Research and Development (PhaReD) Foundation
	Réseau Médicaments et Développement (ReMeD)
	Safe Medicines India
	Council of Europe-Medicrime convention
Medicine Regulatory Authorities and national bodies	Agence Nationale de Sécurité du Médicament et des Produits de Santé (ANSM)
	Centers for Disease Control and Prevention
	Central Drugs Standard Control Organization
	US Food and Drug Administration
	Thailand Food and Drug Administration
	Quality Medicine for all (QUAMED)
	Health Action International (HAI)
	Comité National Anti Contrefaçon (CNAC)

The quality of veterinary medicines and their implications for One Health

	Ghana Food and Drugs Authority
	Health Sciences Authority (HSA)
	Pharmacy and Poisons Board Kenya
	Medicines & Healthcare products Regulatory Agency (MHRA)
	National Agency for Food and Drug Administration and Control (NAFDAC)
	Ordre National de Pharmaciens de Cote d'Ivoire
	Tanzania Medicine & Medical Devices Authority (TMDA)
Alert lists and systems	Pharmabiz
	Association Développement et Santé Contrefaçon Riposte
	mPedigree
	PharmaSecure
	Sproxil
	Les Entreprises du Médicament (LEEM)
	Pfizer Pharmaceutical News
	The Pharmaceutical Security Institute (PSI)
	Sanofi
Newspaper websites with interest in medicine quality	https://www.modernghana.com/
	https://tribuneonlineng.com/
	https://www.allafrica.com/
	https://www.monitor.co.ug/
	https://www.ghanaweb.com/
Other websites with interest in veterinary medicine	https://healthforanimals.org/
	https://www.animalhealtheurope.eu/
	https://www.lepointveterinaire.fr/
