# Supplementary material for: The quality of veterinary medicines and their implications for One Health
Source: BMJ Glob Health. 2022 Aug 1;7(8):e008564. doi: 10.1136/bmjgh-2022-008564 (PMC9351321; doi:10.1136/bmjgh-2022-008564)
Supplement: Supplementary data [file bmjgh-2022-008564supp005.pdf]

The quality of veterinary medicines and their implications for One Health

Supplemental material 5. Number of publication, data points and samples per study/report type in publications (either scientific studies or other reports) containing description of the quality of veterinary medicines in data point(s), i.e. in a specific location at a specific time

	Study type	No. publications n (%)	No. data points n (%)	No samples n (%)
**Scientific reports**	Analysis technique development/validation	24 (12.1%)	36 (6.6%)	79 (0.2%)
	Prevalence survey	20 (10.1%)	173 (31.9%)	1,246 (3.5%)
	Quality control	16 (8.1%)	91 (16.8%)	34,202 (95.7%)
	Equivalence study	11 (5.6%)	28 (5.2%)	188 (0.5%)
	Stability study	2 (1.0%)	3 (0.6%)	14 (0.0%)
	Others	2 (1.0%)	4 (0.7%)	
	**Total**	**75 (37.9%)**	**335 (61.7%)**	**35,729**
**Other reports**	Recall/warning/alert	73 (36.9%)	121 (22.3%)	
	Seizure	48 (24.2%)	85 (15.7%)	
	Case reports	2 (1.0%)	2 (0.4%)	4
	**Total**	**123 (62.1%)**	**208 (38.3%)**	
**Total**		**198 (100.0%)**	**543 (100.0%)**	**35,733**
