# Supplementary material for: The quality of veterinary medicines and their implications for One Health
Source: BMJ Glob Health. 2022 Aug 1;7(8):e008564. doi: 10.1136/bmjgh-2022-008564 (PMC9351321; doi:10.1136/bmjgh-2022-008564)
Supplement: Supplementary data [file bmjgh-2022-008564supp006.pdf]

The quality of veterinary medicines and their implications for One Health

Supplemental material 6. Characteristics of prevalence surveys of veterinary medicines

**Reference/ country of collection**	**Active pharmaceutical ingredients (API)**	**Brand name**	**Outlet/Sampling type**	**Reference specifications standard**	**Test(s) performed and analytical technique for API content/Impurities**	**Failure frequency % (n/N)**
**Farah, 2016 [1]**<br>**Unknown**	Ciprofloxacin	Unknown	Online retrial/ Unknown	USP	API content, Weight uniformity<br><br>HPLC	80.0% (4/5)
**Dicko, 2010 [2]**<br>**Mali**	Oxytetracycline	Unknown	Unknown/ Unknown	International Pharmacopeia<br>European Pharmacopeia<br>USP	API identification, API content, sterility test, pH measurement.<br><br>TLC, UV-visible spectrophotometry	10.8% (13/120)
**Greter, 2016 [3]**<br>**Chad**	Albendazole	Albaneet<br>Alben (green)<br>Alben (white)<br>Albendazole<br>Albenor<br>Benzal<br>Ceva Vermitan NB<br>LOBS Alben	Village markets, health centres, unlicensed outlets, private pharmacies and veterinary pharmacies/Convenience	Conduct of analysis as per Pharmacopoeial status stated on label	API content<br><br>HPLC-UV	46.7% (7/15)
**Ndayisenga, 2009 [4]**<br>**Rwanda**	Albendazole<br>Diminazen-Phenazone<br>Isometamidium<br>Ivermectin<br>Levamisole	Unknown	Import, wholesalers-distributors and veterinary pharmacies/Convenience	European Pharmacopeia	API content, Appearance, Mass uniformity, Disintegration	65.1% (54/83)

The quality of veterinary medicines and their implications for One Health

	Oxytetracycline				HPLC-UV	
**Teko-Agbo, 2003 [5]**<br>**Benin & Togo**	Albendazole<br>Diminazen<br>Isometamidium	Unknown	Unlicensed outlets, wholesalers, veterinary clinic/Random	European Pharmacopeia	API content, Appearance, Disintegration, pH measurement, mass uniformity<br><br>HPLC, UV-Spectrometry	33.3% (12/36)
**Tekle, 2018 [6]**<br>**Ethiopia**	Isometamidium<br>Diminazen	Unknown	Unlicensed outlet, wholesalers, government clinic, private veterinary pharmacy/Convenience	Conduct of analysis as per Pharmacopoeial status stated on label	API content<br><br>HPLC	28.0% (14/50)
**Teko-Agbo, 2009 [7]**<br>**Cameroun & Senegal**	Albendazole<br>Diminazen<br>Ivermectine<br>Levamisole<br>Oxytetracycline	Unknown	Wholesalers, veterinary clinics and pharmacies, unlicensed outlets/ Random	European Pharmacopeia	API content, Appearance, mass uniformity, pH measurement, disintegration<br><br>HPLC	73.8% (76/103)
**Adu, 2016 [8]**<br>**Ghana**	Oxytetracycline<br>Praziquantel	Unknown	Farms, veterinary hospitals, facilities providing medicines (veterinary shops) and manufacturer/Convenience	BP 2013<br>USP 35	API content<br><br>HPLC-UV	52.0% (13/25)
**Assoumy, 2009 [9]**<br>**Ivory coast**	Albendazole<br>Diminazen<br>Isometamidium<br>Ivermectine	Unknown	Wholesalers, distributors and unlicensed outlets/Random and convenience	European Pharmacopeia	API content, mass uniformity, disintegration	43.0% (34/79)

The quality of veterinary medicines and their implications for One Health

	Oxytetracycline				HPLC-UV	
**Tettey, 2001 [10]**<br><br>**Angola**<br>**CAF**<br>**DR Congo**<br>**Ivory Coast**<br>**Malawi**<br>**Mali**<br>**Mozambique**<br>**Namibia**<br>**Nigeria**<br>**Senegal**<br>**Togo**	Diminazen-Phenazone	Berenil<br>Diamyl<br>Diminal<br>Diminaphen<br>Diminasan<br>Diminaveto<br>Diminaze<br>Diminazen<br>Nortryp<br>Nozomil<br>Samorenil<br>Sangavet<br>Trypamyl<br>Trypan<br>Trypazen<br>Veriben<br>Veriben solution	Private pharmacies, veterinary clinics, unlicensed outlets and government supply/ Unknown	BP	API content<br><br>HPLC-DAD	48.5% (50/103)
**Vougat, 2017 [11]**<br><br>**Cameroon**	Oxytetracycline<br>Levamisole<br>Procaine-penicillin G<br>Diminazen	Oxy5%<br>Oxytet[5]<br>Faroxy5%<br>Bolumisole 1<br>Bolumisole 3<br>Fortified Procaine Penicillin<br>Permacain<br>Veriben<br>Diminakel Plus	Unlicensed outlets/ Convenience	Conduct of analysis as per Pharmacopoeial status stated on label.	API content, contaminants<br><br>HPLC, GC/MS	96.3% (26/27)

The quality of veterinary medicines and their implications for One Health

**Phu, 2015 [12]**<br>**Vietnam**	Sulfamethoxazole-trimethoprim<br>Sulfadiazine-trimethoprim<br>Doxycycline-florfenicol<br>Florfenicol-cefalexin<br>Florfenicol-amoxicillin<br>Amoxicillin-ciprofloxacin<br>Florfenicol<br>Doxycycline<br>Amoxicillin	Unknown	Chemical shops/ Convenience	Conduct of analysis as per Pharmacopoeial status stated on label.	API content<br><br>UPLC-MS/MS, HPLC-UV	78.1% (25/32)
**Tran, 2017 [13]**<br>**Vietnam**	Enrofloxacin-ciprofloxacin-amoxicillin<br>Enrofloxacin-oxytetracycline-florfenicol<br>Oxytetracycline-sulfadimethoxin-ormetoprim<br>Sulfamethoxazole-trimethoprim-rifamcine<br>Streptomycin-neomycin-allicin<br>Doxycycline-Oxytetracycline<br>Enrofloxacin-Oxytetracycline<br>Sulfadimethoxin-ormetoprim	Unknown	Chemical shops/ Convenience	Conduct of analysis as per Pharmacopoeial status stated on label.	API content<br><br>UPLC-MS/MS	92.9% (26/28)

The quality of veterinary medicines and their implications for One Health

	Sulfamethoxazole-trimethoprim<br>Doxycycline-rifamcine<br>Erythromycin-rifamcine<br>Sulfadiazine-trimethoprim<br>Ciprofloxacin<br>Oxytetracycline<br>Neomycin<br>Florfenicol					
**Shamaki, 2018 [14]**<br>**Nigeria**	Oxytetracycline<br>Sulphadiazine-trimethoprim<br>Fortified procaine penicillin<br>Albendazole	Unknown<br>Vetcotrim	Veterinary shops/ Convenience	BP<br>USP	API content<br><br><u>UV-visible spectrophotometry</u>	25.0% (1/4)
**Bengaly, 2018 [15]**<br>**Benin**<br>**Burkina Faso**<br>**Ivory Coast**<br>**Mali**<br>**Niger**<br>**Togo**	Diminazen<br>Isometamidium	Unknown	Official: wholesalers, veterinary clinics and veterinary pharmacies<br>Unofficial sources: local market street vendors/ Unknown	World organization for animal health (OIE) monograph	API content<br><br><u>HPLC</u>	47.1% (146/310)
**Li, 2018 [16]**<br>**China**	Amoxicillin<br>Enrofloxacin<br>Florfenicol<br>Sulphadiazine-trimethoprim	Unknown	Chemical supply shops/ Random	Veterinary pharmacopoeia of the people's republic of China	API content, API screening<br><br><u>HPLC-PDA, UPLC-MS/MS</u>	76.4% (54/71)

The quality of veterinary medicines and their implications for One Health

	Sulphamonomethoxine					
**Ratalata, 2008 [17]**<br>**Madagascar**	Oxytetracycline<br>Albendazole<br>Levamisole<br>Closantel	Unknown	Wholesalers, veterinary pharmacies, distributors, unlicensed outlets/ Unknown	European Pharmacopeia	API content, appearance, pH measurement, disintegration, mass uniformity<br>HPLC-UV	57.9% (33/57)
**Yen, 2019 [18]**<br>**Vietnam**	Oxytetracycline-Colistin<br>Amoxicillin-Colistin<br>Oxytetracycline-Streptomycin<br>Thiamphenicol-Sulfamethoxazole<br>Tylosin-Gentamicin<br>Doxycycline-Tylosin<br>Erythromycin-Sulfamethoxazole<br>Gentamicin-Tylosin<br>Tilmicosin<br>Enrofloxacin<br>Amoxicillin-Tylosin<br>Oxytetracycline<br>Neomycin-Colistin<br>Trimethoprim-Colistin	Unknown	Veterinary drug shops/ Convenience	Conduct of analysis as per Pharmacopoeial status stated on label.	API content<br>UPLC-MS/MS	69.5% (41/59)
**Ud Din, 2017 [19]**<br>**Pakistan**	Doxycycline-Tylosin	Unknown	Unknown/ Convenience	BP	API content<br>HPLC-UV	50.0% (4/8)
**Leung, 2020 [20]**	Florfenicol<br>Oxytetracycline	Unknown	Unknown/ Convenience	Conduct of analysis as per Pharmacopoeial	API content	48.4% (15/31)

The quality of veterinary medicines and their implications for One Health

**Hong Kong**				status stated on label.	HPLC	
Note: HPLC: High-performance liquid chromatography; TLC: Thin-layer chromatography; HPLC-UV: High-performance liquid chromatography with UV detector; HPLC-DAD: High-performance liquid chromatography with diode-array detector; GC/MS: Gas chromatography-mass spectrometry; UPLC-MS/MS: Ultra-performance liquid chromatography-tandem mass spectrometer HPLC-PDA: High-performance liquid chromatography with photodiode-array detector<br>USP: United States Pharmacopeia; BP: British Pharmacopeia						

The quality of veterinary medicines and their implications for One Health

REFERENCES

1 Farah C, D'Aoust PM, Krantis A, *et al.* Analysis of ciprofloxacin in fish antibiotic products available from the Internet. *PeerJ Prepr* 2016;**4**:e1856v1. doi:10.7287/peerj.preprints.1856v1

2 Dicko SAA. *La qualité en 2009 des Oxytétracyclines injectables à usage vétérinaire disponibles au Mali*. 2010.http://www.keneya.net/fmpos/theses/2010/pharma/pdf/10P69.pdf (accessed 4 Jul 2018).

3 Greter H, Cowan N, Ngandolo BN, *et al.* Treatment of human and livestock helminth infections in a mobile pastoralist setting at Lake Chad: attitudes to health and analysis of active pharmaceutical ingredients of locally available anthelminthic drugs. *Acta Trop* 2017;**175**:1–9. doi:http://dx.doi.org/10.1016/j.actatropica.2016.05.012

4 Ndayisenga F. *Analyse de la distribution et de la qualité des médicaments vétérinaires au Rwanda*. 2009.http://www.beep.ird.fr/collect/eismv/index/assoc/TD09-37.dir/TD09-37.pdf (accessed 28 Mar 2014).

5 Teko-Agbo A, Biaou FC, Akoda K, *et al.* Fake and defective trypanocidal drugs based on diminazene and anthelminthics formulated with albendazole in Benin and Togo. *Rev Africaine Sante Prod Anim* 2003;**1**:39–47.

6 Tekle T, Terefe G, Cherenet T, *et al.* Aberrant use and poor quality of trypanocides: a risk for drug resistance in south western Ethiopia. *BMC Vet Res* 2018;**14**:1–8. doi:https://dx.doi.org/10.1186/s12917-017-1327-6

7 Teko-Agbo A, AkodaA K, Assoumy AM, *et al.* Quality of veterinary medicinal products in circulation in Cameroon and Senegal. *Dakar Med* 2009;**54**:226–34.

8 Adu AO. *Quality assessment of veterinary pharmaceuticals: a case of oxtetracycline and praziquantel*. 2016.http://ir.knust.edu.gh/bitstream/123456789/9031/1/ABENAA OWUSUWAA ADU.pdf (accessed 10 Aug 2018).

9 Assoumy AM. *Contribution à la codification, à l'analyse des statistiques d'importation et à l'étude de qualité des médicaments vétérinaires en Côte d'Ivoire: cas d'Abidjan, d'Anyama et de Bingerville*. 2009.http://www.beep.ird.fr/collect/eismv/index/assoc/TD09-17.dir/TD09-17.pdf (accessed 2 Aug 2019).

10 Tettey JNA. Report on the investigation of the quality of diminazene preparations in sub-Saharan Africa. 2001.

11 Ngom RRB V, Tomdieu T, Ziebe R, *et al.* Quality of veterinary pharmaceuticals and their use by pastoralists in the Far North Region of Cameroon. *Pastoralism* 2017;**7**:1–14. doi:10.1186/s13570-017-0081-5

12 Phu TM, Phuong NT, Scippo ML, *et al.* Quality of Antimicrobial Products Used in Striped Catfish (Pangasianodon hypophthalmus)

The quality of veterinary medicines and their implications for One Health

Aquaculture in Vietnam. *PLoS One* 2015;**10**:1–8. doi:10.1371/journal.pone.0124267

13 Tran KC, Tran MP, Phan T Van, *et al.* Quality of antimicrobial products used in white leg shrimp (Litopenaeus vannamei) aquaculture in Northern Vietnam. *Aquaculture* 2018;**482**:167–75. doi:10.1016/j.aquaculture.2017.09.038

14 Shamaki BUU, Salihu SI, Daniel N, *et al.* Assessing the Quality of Selected Veterinary Drugs in Nigerian Markets. *Niger J Anim Sci Technol* 2018;**1**:118–24.

15 Bengaly Z, Vitouley SH, Somda MB, *et al.* Drug quality analysis of isometamidium chloride hydrochloride and diminazene diaceturate used for the treatment of African animal trypanosomosis in West Africa. *BMC Vet Res* 2018;**14**:1–8. doi:10.1186/s12917-018-1633-7

16 Li K, Liu L, Zhan J, *et al.* Evaluation of antimicrobial products used in tilapia (Oreochromis spp.) and whiteleg shrimp (Litopenaeus vannamei) aquaculture. *Aquac Res* 2019;**50**:925–33. doi:10.1111/are.13967

17 Ratalata FHR. *Etude de la gestion et de la qualite des medicaments veterinaires en afrique : Cas de Madagacar*. 2008;**8**.http://www.beep.ird.fr/collect/eismv/index/assoc/TD13-3.dir/TD13-3.pdf (accessed 24 May 2019).

18 Yen NTP, Phu DH, Van Nguyen C, *et al.* Labelling and quality of antimicrobial products used in chicken flocks in the Mekong Delta of Vietnam. *Vet Med Sci* 2019;**5**:1–5. doi:10.1002/vms3.189

19 Ud Din R, Khan S, Imtiaz N, *et al.* Study on quality and efficacy of commercial tylosin and doxycycline products against local isolates of mycoplasma in broilers. *Pak J Pharm Sci* 2017;**30**:541–9.

20 Leung KC, Huang Q, St-Hilaire S, *et al.* Fraudulent antibiotic products on the market for aquaculture use. *Prev Vet Med* 2020;**181**:1–5. doi:10.1016/j.prevetmed.2020.105052
