# Supplementary material for: The quality of veterinary medicines and their implications for One Health
Source: BMJ Glob Health. 2022 Aug 1;7(8):e008564. doi: 10.1136/bmjgh-2022-008564 (PMC9351321; doi:10.1136/bmjgh-2022-008564)
Supplement: Supplementary data [file bmjgh-2022-008564supp007.pdf]

The quality of veterinary medicines and their implications for One Health

Supplemental material 7. Failure frequency per type of quality test performed in prevalence survey

Quality attribute test	Failure frequency % (n/N)
API content	46.6% (481/1,032)
Disintegration	23.1% (31/134)
Package/label/physical appearance inspection	1.1% (8/718)
Impurities/Contaminants/Related substances	0.0% (0/27)
Sterility	0.0% (0/120)
Uniformity of units*	29.3% (54/181)
Other chemical analysis**	11.0% (145/1,322)
Unknown/not detailed***	49.5% (105/212)

*Includes content uniformity, weight/mass uniformity, uniformity of mass, and weight variation.
**Includes identification of APIs, pH measurement, and screening (right/wrong API) using Ultra High Performance Liquid Chromatography Mass Spectrometry (UPLC-MS).
***Different quality tests were stated to be performed but results not broken down by test.

Note: One sample may have been tested for one or more quality tests.
