# Supplementary material for: The quality of veterinary medicines and their implications for One Health
Source: BMJ Glob Health. 2022 Aug 1;7(8):e008564. doi: 10.1136/bmjgh-2022-008564 (PMC9351321; doi:10.1136/bmjgh-2022-008564)
Supplement: Supplementary data [file bmjgh-2022-008564supp008.pdf]

The quality of veterinary medicines and their implications for One Health

Supplemental material 8. Samples containing incorrect API and none of the stated API (A) and samples containing incorrect API and variable amount of the stated API(s) (B) in veterinary medicines in prevalence surveys

A. Samples in which incorrect API(s) only were identified (no stated API identified)

Reference	Stated API	% of incorrect API identified (w/w) (API stated on the label not found)	Number of samples
[1]	Florfenicol 10%	0.1%* of sulfadimidine	2
	Florfenicol 10%	8.7%* of enrofloxacin	3
	Florfenicol 20%	6.4 – 6.5%* of enrofloxacin	3
	Florfenicol 2%	0.6%* of enrofloxacin	3
	Sulfadiazine 16% + Trimethoprim 3.2%	4.8%* of sulfamonomethoxine (trimethoprim was not tested)	3
	Sulfamonomethoxine 10%	3.7%* of enrofloxacin	3
	Sulfamonomethoxine 98%	55.8%* of sulfadimidine	1
[2]	Florfenicol 20% + Amoxicillin 5%	11.3% of cephalexin	1

B. Samples in which incorrect API(s) containing variable content of the stated API(s)

Reference	Stated API	% of correct API quantified (compared to that stated on the label) and % of incorrect API identified (w/w)	Number of samples
[3]	Procaine benzylpenicillin (penicillin G) 627 mg	74.2% of procaine benzylpenicillin (low %API) and Amiodarone**	1
	Procaine benzylpenicillin (penicillin G) 627 mg	135.4% of procaine benzylpenicillin (high %API) and Amiodarone**	1
	Procaine benzylpenicillin (penicillin G) 627 mg	96.0% of procaine benzylpenicillin (right %API) and Amiodarone**	1

The quality of veterinary medicines and their implications for One Health

	Procaine benzylpenicillin (penicillin G) 600 mg	91.2 and 101.7% of procaine benzylpenicillin (right %API) and Amiodarone**	2
	Procaine benzylpenicillin (penicillin G) 600 mg	81.7% of procaine benzylpenicillin (low %API) and Amiodarone**	1
[1]	Amoxicillin 5%	64.0 – 78.0% of amoxicillin (low %API) and 0.6%* of sulfadimidine	3
	Amoxicillin 70%	7.9 – 8.7% of amoxicillin (low %API) and 1.8%* of sulfadimidine	3
	Enrofloxacin 10%	42.0% of enrofloxacin (low %API) and 3.3 – 3.8%* of amoxicillin and 0.4%* of sulfadimidine	3
	Enrofloxacin 5%	144.0 – 146.0% of enrofloxacin (high %API) and 5.8 – 5.9%* of sulfadiazine	3
	Florfenicol 10%	18.0% of florfenicol (low %API) and 0.1%* of sulfadimidine	1
	Florfenicol 5%	30.0% of florfenicol (low %API) and 0.7%* of enrofloxacin and 0.3% of sulfadimidine	1
	Florfenicol 5%	108.0% of florfenicol (right %API) and 14.0%* of enrofloxacin and 6.0% of sulfadimidine	2
	Florfenicol 10%	102.0 – 109.0% of florfenicol (right %API) and 0.5%* of enrofloxacin	3
	Sulfadiazine 3% + Trimethoprim 0.6%	63.3% of sulfadiazine (low %API) and 0.5%* of enrofloxacin (trimethoprim was not tested)	2
[2]	Sulfadiazine 33% + Trimethoprim 6.7%	11.0% of sulfamethazine (no sulfadiazine) and 47.7% of trimethoprim (low %API)<br>10.5% of sulfamethazine (no sulfadiazine) and 32.4% of trimethoprim (low %API)	2
	Florfenicol 40% + Cefalexin 35%	45.5% of florfenicol (low %API) and 10.1% of amoxicillin (no cefalexin)	1

The quality of veterinary medicines and their implications for One Health

[4]	Oxytetracycline 35% + Sulfadimethoxine 30% + Ormetoprim 10%	116.0% of oxytetracycline (high %API) and 0.45% of florfenicol and 10% sulfadiazine (no sulfadimethoxine and ormetoprim)<br>82.0% of oxytetracycline (low %API) and 0.45% of florfenicol and 10% sulfadiazine (no sulfadimethoxine and ormetoprim)	2
	Sulfadiazine 33% + Trimethoprim 7%	1.0% of sulfamethoxazole (no sulfadiazine) and 39.0% of trimethoprim (low %API)	1

Notes:
** We present the percentage of identified incorrect API(s) as it is declared in publications but it is often not clear how the authors calculated the percentage.*
*** The percentage of Amiodarone was not stated by the authors.*

The quality of veterinary medicines and their implications for One Health

References

1 Li K, Liu L, Zhan J, et al. Evaluation of antimicrobial products used in tilapia (Oreochromis spp.) and whiteleg shrimp (Litopenaeus vannamei) aquaculture. *Aquac Res* 2019;50:925–933.

2 Phu TM, Phuong NT, Scippo ML, et al. Quality of Antimicrobial Products Used in Striped Catfish (Pangasianodon hypophthalmus) Aquaculture in Vietnam. *PLoS One* 2015;10:1–8. doi:10.1371/journal.pone.0124267

3 Ngom RRBV, Tomdieu T, Ziebe R, et al. Quality of veterinary pharmaceuticals and their use by pastoralists in the Far North Region of Cameroon. *Pastoralism* 2017;7:1–14. doi:10.1186/s13570-017-0081-5

4 Tran KC, Tran MP, Phan T Van, et al. Quality of antimicrobial products used in white leg shrimp (Litopenaeus vannamei) aquaculture in Northern Vietnam. *Aquaculture* 2018;482:167–175. doi:10.1016/j.aquaculture.2017.09.038
