# Supplementary material for: The quality of veterinary medicines and their implications for One Health
Source: BMJ Glob Health. 2022 Aug 1;7(8):e008564. doi: 10.1136/bmjgh-2022-008564 (PMC9351321; doi:10.1136/bmjgh-2022-008564)
Supplement: Supplementary data [file bmjgh-2022-008564supp009.pdf]

The quality of veterinary medicines and their implications for One Health

Supplemental material 9. Failure frequency of samples collected in prevalence surveys per type of outlet

Type of outlet	Failure frequency % (n/N)
Manufacturer	100.0% (1/1)
Chemical shops*	80.2% (105/131)
Online pharmacy	80.0% (4/5)
Farm*	75.0% (3/4)
Veterinary clinics/hospitals/health centres	60.0% (18/30)
Veterinary medicines outlets	58.9% (43/73)
Combination of outlets	57.0% (298/523)
Unregistered/unlicensed outlets/facilities	45.9% (90/196)
Wholesalers/importer/distributors	43.8% (56/128)
Unknown	19.4% (30/155)
**Total**	**52.0% (648/1,246)**

Note:
**The author did not clarified the definition of outlet type*
