# Supplementary material for: The quality of veterinary medicines and their implications for One Health
Source: BMJ Glob Health. 2022 Aug 1;7(8):e008564. doi: 10.1136/bmjgh-2022-008564 (PMC9351321; doi:10.1136/bmjgh-2022-008564)
Supplement: Supplementary data [file bmjgh-2022-008564supp010.pdf]

BMJ Publishing Group Limited (BMJ) disclaims all liability and responsibility arising from any reliance placed on this supplemental material which has been supplied by the author(s)

The quality of veterinary medicines and their implications for One Health

Supplemental material 10. Failure frequencies of samples collected in prevalence surveys by region of stated manufacturer

**Continent**	**Failure frequency % (n/N)**
America	77.8% (14/18)
Asia	76.0% (139/183)
Africa	66.7% (4/6)
Europe	64.4% (67/104)
Unknown	45.3% (424/935)
**Total**	**52.0% (648/1,246)**
