# Supplementary material for: The quality of veterinary medicines and their implications for One Health
Source: BMJ Glob Health. 2022 Aug 1;7(8):e008564. doi: 10.1136/bmjgh-2022-008564 (PMC9351321; doi:10.1136/bmjgh-2022-008564)
Supplement: Supplementary data [file bmjgh-2022-008564supp011.pdf]

The quality of veterinary medicines and their implications for One Health
Supplemental material 10. Frequency of agreement with the 26 items of the MEDQUARG checklist of the 15 prevalence surveys

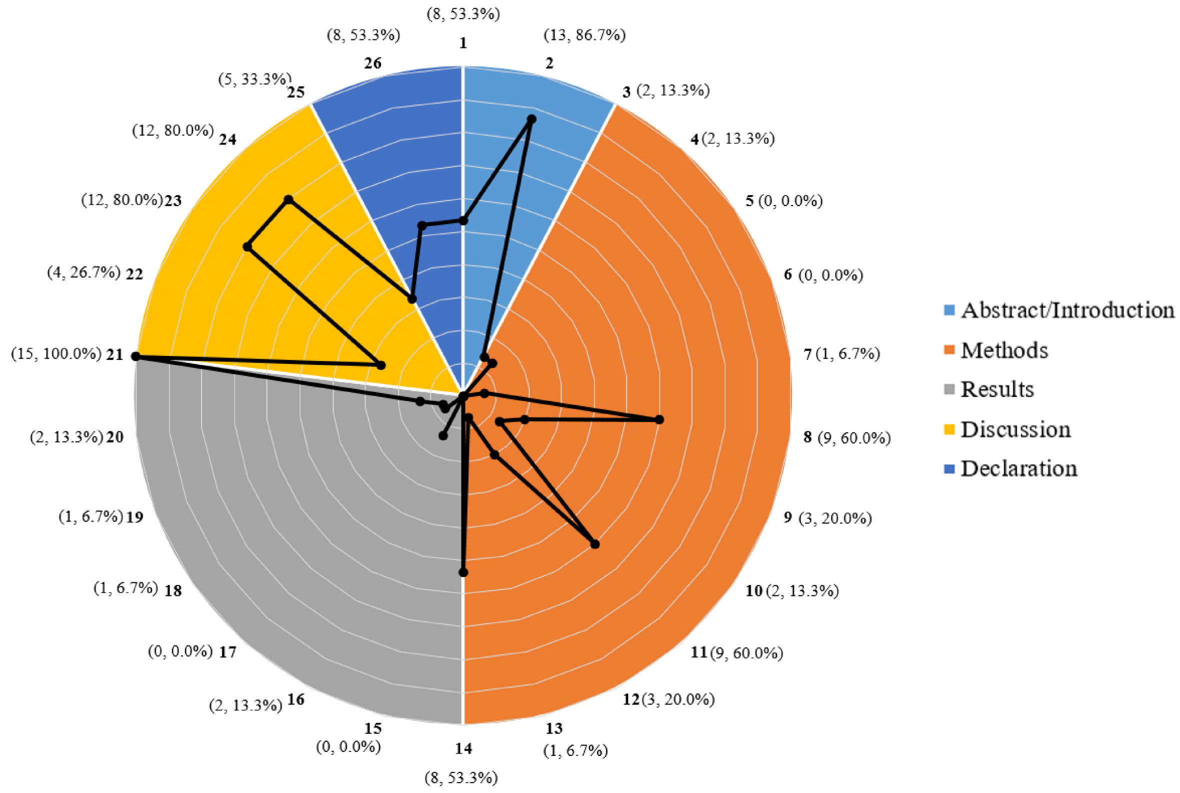

Definition of items: 1. Title/abstract/keywords 2. Introduction.
Methods (items 3-13): 3. Survey details 4. Definitions 5. Outlets 6. Sampling design 7. Samplers 8. Statistical methods 9. Ethical issues 10. Packaging 11. Chemical analysis 12. Method validation 13. Blinding.
Results (items 14-20): 14. Outlets (actual) 15. Missing samples 16. Packaging and chemistry results 17. Category of poor quality medicine 18. State company and address as given on packaging 19. Sharing data with MRA 20. Dissemination.
Discussion (items 21-24): 21. Key results 22. Limitations 23. Interpretation 24. Intervention.
Declaration: 25. Conflict of interest 26. Funding.

The quality of veterinary medicines and their implications for One Health

No	Item	Tettey, JNA. et al. 2001	Teko-Agbo, A. et al. 2003	Teko-Agbo, A. et al. 2009	Phu TM. et al. 2015	Farah, C. et al. 2016	Adu, AO. et al. 2016	Vougat Ngom, RRB. et al. 2017
**1**	Title/abstract/keywords	N	N	Y	Y	N	N	N
**2**	Introduction	Y	Y	Y	N	Y	Y	Y
**Methods**								
**3**	Survey details	N	Y	Y	N	N	N	N
**4**	Definitions	N	N	N	N	N	N	N
**5**	Outlets	N	N	N	N	N	N	N
**6**	Sampling design	N	N	N	N	N	N	N
**7**	Samplers	N	N	N	N	N	N	Y
**8**	Statistical methods	N	Y	Y	N	N	N	Y
**9**	Ethical issues	N	N	N	N	N	N	N
**10**	Packaging	N	Y	Y	N	N	N	N
**11**	Chemical analysis	Y	Y	Y	Y	N	Y	N
**12**	Method validation	Y	N	N	N	N	Y	N
**13**	Blinding	N	N	N	N	N	N	N
**Results**								
**14**	Outlets	Y	N	N	Y	Y	Y	Y
**15**	Missing samples	N	N	N	N	N	N	N
**16**	Packaging and chemistry results	N	N	N	N	Y	N	Y
**17**	Category of poor-quality medicine	N	N	N	N	N	N	N
**18**	State company and address as given on packaging	Y	N	N	N	N	N	N
**19**	Sharing data with MRA	N	N	N	N	N	N	N
**20**	Dissemination	N	N	N	N	N	Y	Y
**Discussion**								
**21**	Key results	Y	Y	Y	Y	Y	Y	Y
**22**	Limitations	N	Y	Y	N	Y	N	Y
**23**	Interpretation	Y	Y	Y	Y	N	Y	Y

The quality of veterinary medicines and their implications for One Health

**24**	Intervention	Y	Y	Y	Y	N	Y	N
**Declaration**								
**25**	Conflict of interest	N	N	N	Y	N	N	Y
**26**	Funding	N	N	N	Y	N	N	Y
	**Total score and percentage**	8	9	10	8	5	8	11
		30.8%	34.6%	38.5%	30.8%	19.2%	30.8%	42.3%

Note: Only the prevalence surveys published as original articles in scientific journals or following the Introduction/Methods/Results/Discussion or similar style and published as reports or PhD thesis, were appraised.

Y: reported, N: not reported

The quality of veterinary medicines and their implications for One Health

No	Item	Tran, KC. et al. 2017	Riaz ud Din et al. 2017	Tekle, T. et al. 2018	Shamaki, BU. et al. 2018	Bengaly, Z. et al. 2018	Li, K. et al. 2018	Yen, NTP. et al. 2019	Leung, K. C. et al. 2020
**1**	Title/abstract/keywords	Y	Y	N	Y	Y	N	Y	Y
**2**	Introduction	Y	Y	Y	Y	Y	Y	N	Y
**Methods**									
**3**	Survey details	N	N	N	N	N	N	N	N
**4**	Definitions	Y	Y	N	N	N	N	N	N
**5**	Outlets	N	N	N	N	N	N	N	N
**6**	Sampling design	N	N	N	N	N	N	N	N
**7**	Samplers	N	N	N	N	N	N	N	N
**8**	Statistical methods	N	Y	Y	Y	Y	N	Y	Y
**9**	Ethical issues	N	N	Y	N	Y	N	Y	N
**10**	Packaging	N	N	N	N	N	N	N	N
**11**	Chemical analysis	Y	Y	Y	N	Y	N	N	N
**12**	Method validation	N	N	N	N	N	Y	N	N
**13**	Blinding	N	N	Y	N	N	N	N	N
**Results**									
**14**	Outlets	Y	N	Y	N	Y	N	N	N
**15**	Missing samples	N	N	N	N	N	N	N	N
**16**	Packaging and chemistry results	N	N	N	N	N	N	N	N
**17**	Category of poor-quality medicine	N	N	N	N	N	N	N	N
**18**	State company and address as given on packaging	N	N	N	N	N	N	N	N
**19**	Sharing data with MRA	N	N	N	Y	N	N	N	N
**20**	Dissemination	N	N	N	N	N	N	N	N
**Discussion**									
**21**	Key results	Y	Y	Y	Y	Y	Y	Y	Y
**22**	Limitations	N	N	N	N	N	N	N	N
**23**	Interpretation	Y	Y	Y	Y	N	Y	N	Y
**24**	Intervention	N	Y	Y	Y	Y	Y	Y	Y
**Declaration**									

The quality of veterinary medicines and their implications for One Health

**25**	Conflict of interest	N	N	Y	N	Y	N	Y	N
**26**	Funding	Y	N	Y	N	Y	Y	Y	Y
**Total score and percentage**		8	8	11	7	10	6	7	7
		30.8%	30.8%	42.3%	26.9%	38.5%	23.1%	26.9%	26.9%

Note: Only the prevalence surveys published as original articles in scientific journals or following the Introduction/Methods/Results/Discussion or similar style and published as reports or PhD thesis, were appraised.

Y: reported, N: not reported
