# Supplementary material for: The quality of veterinary medicines and their implications for One Health
Source: BMJ Glob Health. 2022 Aug 1;7(8):e008564. doi: 10.1136/bmjgh-2022-008564 (PMC9351321; doi:10.1136/bmjgh-2022-008564)
Supplement: Supplementary data [file bmjgh-2022-008564supp012.pdf]

The quality of veterinary medicines and their implications for One Health

Supplemental material 12. Characteristics of equivalence surveys of veterinary medicines quality

**Reference / country of collection**	**Active pharmaceutical ingredient (API)**	**Brand name**	**Outlet types**	**Reference standard**	**Test(s) performed and analytical technique for API content/Impurities**	**Failure frequency % (n/N)**
**Tettey, 1999 [1]**<br>**United Kingdom**	Isometamidium	Samorin®<br>Veridium®	Unspecified	Unspecified	API content<br>HPLC-UV	0.0% (0/8)
**Scott-Moncrieff, 2012** [2]<br>**United states of America**	Protamine zinc insulin	Compounded product (n=12) and unknown brand name (n=2)	Compounding company and manufacturers	USP	API content, zinc concentration, pyrogenicity test, pH, appearance, crystal size, insulin concentrations in the supernatant.<br>HPLC	78.6% (11/14)
**Saba, 2012 [3]**<br>**Nigeria**	Oxytetracycline	Unknown brand name	Veterinary pharmacies	Unspecified	API concentrations<br>Spectrophotometer	100.0% (12/12)
**Cook, 2012 [4]**<br>**United States of America**	Trilostane	Compounded product	Pharmacies	USP	API content, impurities test, dissolution test<br>HPLC-PAD	37.5% (36/96)

The quality of veterinary medicines and their implications for One Health

**Vidal, 2013 [5]**<br>**Uruguay**	Albendazole	Unknown brand name	Veterinary shops	USP	API content, dissolution test<br><br>HPLC-UV-Visible	33.3% (1/3)
**Gberindyer, 2014 [6]**<br>**Nigeria**	Albendazole	Unknown brand name	Local veterinary shops	USP 2007	Weight uniformity, API content, hardness, friability, disintegration, dissolution<br><br>UV-spectrophotometer	100.0% (9/9)
**Laporte, 2017 [7]**<br>**United states of America**	Fluconazole	Compounded product	Veterinary pharmacies	USP	API content, impurities, pH, appearance<br><br>HPLC-UV	6.3% (1/16)
**Gberindyer, 2017 [8]**<br>**Nigeria**	Gentamicin sulphate	Generic	Local veterinary and pharmaceutical stores	USP	API content<br><br>HPLC-UV-Visible	12.5% (1/8)
**Gberindyer, 2019 [9]**<br>**Nigeria**	Amoxicillin-Gentanicin<br>Amoxicillin-Colistin	Unknown brand name	Pharmaceutical and veterinary shops and clinics	USP, IP	API content<br><br>HPLC-UV	70.0% (7/10)

The quality of veterinary medicines and their implications for One Health

**Seifu, 2019 [10]**<br>**Ethiopia**	Albendazole	Unknown brand name	Veterinary pharmacies	USP	API identification, API content, appearance, weight uniformity<br><br><u>UV-visible spectroscopy, FTIR, HPLC-UV-Visible</u>	60.0% (6/10)
**Waller, 2020 [11]**<br>**Brazil**	Itraconazole	Unknown brand name (n=1) and compounded product (n=1)	Veterinary pharmacies and compounding pharmacies	Manufacturer specification	API content, antifungal efficacy<br><br><u>HPLC-MS,</u><br><u>In-vitro antifungal susceptibility assay</u>	100.0% (2/2)

The quality of veterinary medicines and their implications for One Health

References:

1 Tettey JNA, Skellern GG, Grant MH, et al. Investigation of the chemical equivalence of the trypanocidal products, Samorin® and Veridium®. *J Pharm Biomed Anal* 1999;21:1–7. doi:10.1016/S0731-7085(99)00110-7

2 Scott-Moncrieff JC, Moore GE, Coe J, et al. Characteristics of commercially manufactured and compounded protamine zinc insulin. *J Am Vet Med Assoc* 2012;240:600–605. doi:10.2460/javma.240.5.600

3 Saba AB, Olatoye IO, Oridupa OA. Spectrophotometric analysis of oxytetracycline brands available over-the-counter for veterinary use in south western Nigeria. *Niger Vet J* 2012;33:523–530.http://www.ajol.info/index.php/nvj/article/view/86427

4 Cook AK, Nieuwoudt CD, Longhofer SL. Pharmaceutical evaluation of compounded trilostane products. *J Am Anim Hosp Assoc* 2012;48:228–233. doi:10.5326/jaaha-ms-5763

5 Vidal NLG, Starkloff WJ, Bentancor S, et al. Comparative dissolution studies of Albendazole oral suspensions for veterinary use. *Dissolution Technol* 2013;20:27–30. doi:http://dx.doi.org/10.14227/DT200413P27

6 Gberindyer FA, Onyeyili PA, Bosha JA. Quality Control Properties of Some Brands of Veterinary Albendazole Boluses Common in Nigeria. *J Pharm Pharmacol* 2014;2:135–139.

7 Laporte CM, Cruz-Espindola C, Thungrat K, et al. Quality assessment of fluconazole capsules and oral suspensions compounded by pharmacies located in the United States. *Am J Vet Res* 2017;78:421–432. doi:https://dx.doi.org/10.2460/ajvr.78.4.421

8 Gberindyer FA, Matthew Oluwole Abatan, Lubbe Wiesner. Composition of Gentamicin C Components in Gentamicin Sulphate Generics Commonly Used in Small Animal Practice in Nigeria. *J Pharm Pharmacol* 2017;5:20–25. doi:10.17265/2328-2150/2017.01.005

9 Gberindyer FA, Charles OS, Moshood ARA, et al. Evaluation of amoxicillin content in commonly used multisource injectable brands in veterinary practice. *Egypt J Vet Sci* 2019;50:159–165. doi:10.21608/ejvs.2019.13433.1082

10 Seifu A, Kebede E, Bacha B, et al. Quality of albendazole tablets legally circulating in the pharmaceutical market of Addis Ababa, Ethiopia: physicochemical evaluation. *BMC Pharmacol Toxicol* 2019;20:7. doi:10.1186/s40360-019-0299-5

11 Waller SB, Ripoll MK, Madrid IM, et al. Susceptibility and resistance of Sporothrix brasiliensis to branded and compounded itraconazole formulations. *Braz J Microbiol* Published Online First: 2020. doi:http://dx.doi.org/10.1007/s42770-020-00280-7
