# Supplementary material for: The quality of veterinary medicines and their implications for One Health
Source: BMJ Glob Health. 2022 Aug 1;7(8):e008564. doi: 10.1136/bmjgh-2022-008564 (PMC9351321; doi:10.1136/bmjgh-2022-008564)
Supplement: Supplementary data [file bmjgh-2022-008564supp013.pdf]

The quality of veterinary medicines and their implications for One Health

Supplemental material 13. Detailed results for the equivalence studies (n= 11) of veterinary medicines

In 11 equivalence studies, most of samples were tested for more than one quality attribute (85.1%, n= 160). All 188 samples were tested for API identification and API content, 118 samples (62.8%) for impurities/contaminant and 108 samples (57.4%) for dissolution test. Packaging analysis was conducted for 13.9% (n= 26) of 188 samples.

The failure frequency due to API content test failure (43.1%, 81/188) was the highest, followed by other physical tests (42.6%, 46/108) and dissolution test failure (23.1%, 25/108). In one study, out of 96 samples tested for API content, dissolution and impurities, there were no breakdown of the results by test for 60 (31.9%) samples.[1] None of the 26 samples tested for packaging inspection failed.

Of 81 samples that failed API content test, 49.4% (n= 40) contained lower API amount than stated on the packaging and 6.2% (n= 5) higher API. For 44.4% (n= 36) of the samples there was not enough details in the publication as to whether they contained higher or lower amounts of API.

Failure frequency by quality test in equivalence studies

**Quality test**	**Failure frequency % (n/N)**
API content	43.1% (81/188)
Package/label/physical appearance inspection	32.5% (13/40)
Dissolution	23.1% (25/108)
Impurities/contaminants/related substances	1.0% (1/96)
Uniformity of units*	57.9 (11/19)
Other physical analysis**	29.3% (12/41)
Other chemical analysis***	8.0% (18/224)
Other techniques****	0.1% (3/38)

* Includes content uniformity, weight/mass uniformity, uniformity of mass, and weight variation.
**Includes crystal size, disintegration, friability, hardness.
***Includes API identification, pH and zinc content.
****Includes bacterial susceptibility, infectious agents contamination, insulin concentrations in the supernatant and pyrogenicity.

Note: One sample may have been tested for one or more quality tests

Failure frequency by version of drugs in equivalence studies

**Version of drug**	**Failure frequency % (n/N)**
Compounded	50.4% (63/125)
**Generic**	12.5% (1/8)
**Unknown**	67.3% (37/55)

The quality of veterinary medicines and their implications for One Health

**Total**	**53.7% (101/188)**

Reference:

1 Farah C, D'Aoust PM, Krantis A, et al. Analysis of ciprofloxacin in fish antibiotic products available from the Internet. *PeerJ Prepr* 2016;4:e1856v1. doi:10.7287/peerj.preprints.1856v1
