# Supplementary material for: The quality of veterinary medicines and their implications for One Health
Source: BMJ Glob Health. 2022 Aug 1;7(8):e008564. doi: 10.1136/bmjgh-2022-008564 (PMC9351321; doi:10.1136/bmjgh-2022-008564)
Supplement: Supplementary data [file bmjgh-2022-008564supp014.pdf]

The quality of veterinary medicines and their implications for One Health
Supplemental material 14. Recalls, alerts, seizures and case reports of poor-quality veterinary medicines.

Recalls and alerts

Month and Year of publication	Country	API/API combination	No. batches	Description of the incident	Reference
January 2005	Uganda	Isometamidium	1	API content – No API<br>'Farmers should be on the lookout for fake veterinary drugs on the market […]. The test led to the conclusion that Samorin 1g, batch number R 282971 with expiry date 06/2005 does not contain the claimed pharmaceutical ingredient Isometamidium hydrochloride, […]'	[1]
April 2006	Canada	Unspecified	All batches	No marketing authorization<br>'Veterinary drug with no marketing authorization (no DIN).'	[2]
December 2006	Canada	Doxycycline	1	Content uniformity failure<br>'Failure to meet mixing uniformity specification, during routine confirmatory testing.'	[3]
June 2007	China	Unspecified	Unstated	Falsified veterinary medicines – no details on defects<br>'Almost 20% of veterinary drugs tested in China in the early part of 2007 did not pass tests for minimum standards.'	[4]
June 2008	Canada	Neomycin-Sulfamethazine	1	API content – Low API<br>'Lot 2724786 is being recalled due to an out of specification neomycin assay. Results of approximately 82% of label claim (spec. 90-115%) have been confirmed for samples of this lot. This issue was found during an investigation into a product complaint related to the observation of an atypical product appearance (color).'	[5]

The quality of veterinary medicines and their implications for One Health

October 2009	Canada	Benzylpenicillin (penicillin G)	5	Unknown quality defect<br>'FDA Consent Decree of Teva Animal Health'	[6]
January 2010	United States	Ketamine<br>Butorphanol	Unstated	Unknown quality defect<br>'[…] This recall is being conducted as a result of an increased trend in serious adverse events associated with this product, including lack of effect, prolonged effect, and death" (Five cats died) & "Teva Animal Health […] shut down on July 31, [*the FDA*] citing adulterated drugs and multiple violations of good manufacturing practices at its St. Joseph, Mo., facility.'	[7]
July 2011	Canada	Gentamicin-Betamethasone	1	Stability test failure<br>'TOPAGEN Spray, lot 0CFYA05 is being recalled due to an out-of-specification result obtained for the active ingredient Betamethasone valerate content during the ongoing stability testing program at the 12-month time point.'	[8]
November 2011	Canada	Amoxicillin-Clavulanic acid	2	Stability test failure<br>'Through their stability program for Bioclav (375mg tablets) for veterinary use, it has been determined that the product may not meet its intended shelf life'	[9]
October 2012	Canada	Bacitracin-Neomycin-Polymycin B	1	Packaging defect<br>'Break of sterility at the tube fold'	[10]
November – December 2012	Canada	Cetrimide	2	Microbial contamination<br>'Microbial contamination at 3 month stability: *Pseudomonas aeruginosa* for Lot M0327. Due to recall of Lot M0327, retention samples from Lot 1112 were tested and *Pseudomonas aeruginosa* was detected as well.'	[11]
November 2013	Canada	Chloramphenicol	2	Stability test failure<br>'A potential lack of homogeneity was observed in recalled batches of the product that could result in separation of the oil and ointment.'	[12]

The quality of veterinary medicines and their implications for One Health

March 2014	Canada	Bacitracin	All batches	Mislabeling<br>'Some bags may be missing the lot number and expiry date label'	[13]
August 2014	United Kingdom	Meloxicam	Unstated	Falsified– unknown defect<br>'Boehringer Ingelheim warned "that a presence of a counterfeit vial of Metacam injection has been identified in the UK and confirmed by the company".[…] "The counterfeit vial carried a poor copy of the label, was made from a different type of glass, stopper and crimp-on cap and carried a false batch number and expiry date information.'	[14]
April 2015	Canada	Follicle stimulating hormone	1	Product sterility<br>'Recall initiated following an Out of Specification results during sterility testing for vials of Folltropin-V (lot R2B08) contained in the kits of R2B08A & R2B08G lots.'	[15]
November 2015	India	Oxytocin	3	API content – No details on API content failure with pH, sterility failures and presence of particles of unknown origin.<br>'Name of Drugs: Oxytocin Injection Vet, B. No.: Not Mentioned, Mfg dt: Not Mentioned, Exp dt: Not Mentioned, Mfd by: M/s. Not Mentioned; Reason of failure: Description, Particulate Matter, pH & Assay.'<br>'Name of Drugs: Oxytocin Injection Vet, No.: Not Mentioned, Mfg dt: Not Mentioned, Exp dt: Not Mentioned, Manufactured in India by Gaya; Reason of failure: Description, Particulate Matter & Assay.'<br>'Name of drugs: Oxytocin Injection B.P. (Vet); B. No.: 10, Mfg dt: 01/2014, Exp dt: 2 years, Mfd by: M/s. S. Pharmaceuticals, B.O. Kanpur;	[16]

The quality of veterinary medicines and their implications for One Health

				Reason of failure: Acidity, Sterility, Assay & Description.’	
December 2015	India	Oxytocin	1	API content – No details on API content failure with pH failure and presence of particles of unknown origin<br>‘Name of drugs: Oxytocin Injection B.P. (Vet); B. No.: 07, Mfg dt: 03/2015, Exp dt: 02/2017, Mfd by: M/s. Neclo Chemicals Works, Rang Bahadur Road, Gaya; Reason for failure: Description, Paticulate Matter, pH & Assay […]’	[17]
January 2016	India	Oxytocin	1	API content – No details on API content failure with pH failure and presence of particles of unknown origin<br>‘Name of drugs: Oxytocin Injection BP Vet.; B. No.: 19, Mfg dt: 06/2015, Exp dt: 05/2017, Mfd by: M/s. Regain Laboratories, 134/5, HTM Road, Raipur Lane, Hisar 125001; Reason for failure: Description, pH, Paticulate Matter & Assay’	[18]
January 2016	Canada	Tetracycline	2	API content – No details on API content failure<br>‘Out of specification result for degradation product assay during shelf life testing.’	[19]
April 2016	United Kingdom	Isofluran	1	Packaging defect<br>‘Re-sealed bottles may not be airtight because of a defect with the metal cap thread’	[20]
June 2016	India	Amitraz	1	Falsified – API identification and no details on API content failure<br>‘Name of Drugs: Amitraz Dip Concentrate Liquid IP vet. (Amitraz 12.5%); B. No: S-1129, Mfg dt: 01/2016, Exp dt: 12/2018, Mfd by: M/s. Saibliss Drugs & Pharmaceuticals, 66-67, Gondpur Ind. Aream Ponta Sahib-173025; Reason of failure: Identification and Assay.’	[21]
December 2016	United Kingdom	Detomidine	2	Unknown quality defect	[22]

The quality of veterinary medicines and their implications for One Health

				'A high incident of lack of efficacy has been reported for batches: DTH004 and DTH006'	
December 2016	United Kingdom	Meloxicam	1	Mislabeling<br>'[…] The 1ml syringe within the pack has 'Lbs' printed as the unit of measure instead of kg.'	[23]
February 2017	United Kingdom	Levamisole-Triclabendazole	4	Product defect<br>'When the product is used in conjunction with a product applicator gun, the applicator gun fails to operate, causing no product to pass through the gun and be administered to the animal.'	[24]
March 2017	United Kingdom	Propofol	All batches	Packaging defect<br>'An issue of coring has been reported, where the shearing off of a portion of the 20mm bromobutyl bung occurs as the vial is pierced to withdraw the product.'	[25]
May 2017	United States	Phenobarbital<br>Amitriptyline	15<br>1	Mislabeling<br>'[…] due to the initial recall resulting from a label mix-up error, out of an abundance of caution, we are recalling all products […]'	[26]
May 2017	United Kingdom	Cypermethrin	3	Packaging defect<br>'The company has received a number of reports of defective aluminium foil seals in a number of units from the batches of Deosect 5%, resulting in slight leakage of the product during storage.'	[27]
May 2017	United Kingdom	Permethrin	1	Packaging defect<br>'Some units in this batch have been reported to be leaking a small amount of product from around the cap due to the induction heat seals in some packs not being completely sealed to the bottle.'	[28]
August 2017	India	Oxytocin	2	API content – No details on API content failure with pH and sterility failure<br>'Name of Drugs: Oxytocin Injection B.P. (Vet); B. No.: OXY 003, Mfg dt: 12/2013, Exp dt:	[29]

The quality of veterinary medicines and their implications for One Health

				11/2015; Mfd by: M/s. Priya Pharmaceuticals, 823001, B.O. Kanpur.; Reason for failure: Acidity, Sterility and Assay.'<br>'Name of Drugs: Oxytocin Injection B.P. (Vet); B. No.: OXY 003, Mfg dt: 12/2015, Exp dt: 11/2017; Mfd by: M/s. Priya Pharmaceuticals, 823001, B.O. Kanpur.; Reason for failure: Acidity, Sterility and Assay.'	
October 2017	United Kingdom	Meloxicam	2	pH failure<br>'Norbrook have verified that the pH of certain batches has increased over time and is out of specification.'	[30]
February 2018	United Kingdom	Doxycycline	3	Stability test failure<br>'Stability tests have showed that certain bags of the 200g packaging of the product could be damaged minimally.'	[31]
March 2018	United Kingdom	Oxytocin	1	Contained a lower level of the preservative chlorobutanol hemihydrate<br>'[…] there is a potential for decreased levels of the preservative Chlorobutanol hemihydrate.'	[32]
April 2018	United Kingdom	Butorphanol	1	Contamination during production-unspecified<br>'Werfft GmbH has issued a batch recall of Alvegesic vet. 10mg/ml Solution for injection for Horses, Dogs and Cats (Vm 32802/4000) due to contamination during production.'	[33]
June 2018	United Kingdom	Azaperone	1	Contamination during production-unspecified<br>'Elanco has identified that inconsistencies in the cleaning process have led to low level of a previous product being carried over into the Stresnil batch'	[34]
June 2018	Canada	Propofol	1	Mislabeling<br>'A portion of the affected lot is packaged with an incorrect label.'	[35]

The quality of veterinary medicines and their implications for One Health

June 2018	United Kingdom	Cypermethrin	3	Packaging defect<br>'This recall is due to the possible presence of a defect in the pack, which may cause the cap to split.'	[36]
July 2018	United Kingdom	Unspecified	4	Product defect – no details on defects	[37]
July 2018	United Kingdom	Rabbit Haemorrhagic Disease Virus vaccine	2	Storage problem- unspecified<br>'The following batches of Filavac VHD K C+V (Vm 46470/4000) have been recalled due to incorrect storage requirements during shipment.'	[38]
July 2018	United Kingdom	Procaine-Epinephrine	6	Stability test failure<br>'The following batches of Willcain Solution for Injection (Vm 10434/4046) have been recalled due to low active substance assay results during long term stability trial.'	[39]
July 2018	Rwanda	Unspecified	Unstated	Falsified and expired veterinary medicines – no details on defects.<br>'Drugs worth about Rwf24m have been impounded, four animal drug stores closed and three people arrested in an operation that was intended to tackle sale and use of counterfeit veterinary products in the country.' […] '18 of the pharmacies were found to be selling drugs that are illegal, with some being counterfeit, others not allowed in the country, while others were found to have expired.[…]'	[40]
August 2018	Canada	Kaolin-Pectin	4	Product sterility<br>'Presence of mold in the affected lots'	[41]
November 2018	United Kingdom	Carprofen	1	Product sterility<br>'Norbrook Laboratories Ltd is recalling the below mentioned batch from the market place as a precautionary measure as sterility cannot be assured.'	[42]
January 2019	United Kingdom	Ivermectin-Praziquantel	2	Packaging defect	[43]

The quality of veterinary medicines and their implications for One Health

				'Packs of 48 syringes each containing 7.49g of product are being recalled with immediate, as they do not contain English language package leaflets and the immediate packaging (sticker on dosing syringe) is not in English.'	
March 2019	United States	Pilocarpine	1	Contained a higher level of the preservative 'benzalkonium chloride'.<br>'The ophthalmic solution has been found to contain a higher level of the preservative benzalkonium chloride than is typical.'	[44]
March 2019	United States	Enrofloxacin<br>Ivermectin	2<br>4	Product sterility<br>'There is a concern that if the sterility of these products has been compromised, use of these products could result in introduction of infectious agents to the animal.'	[45]
March 2019	United Kingdom	Ketamine	Unstated	Presence of particles of unknown origin<br>'[…] Le Vet Pharma has issued a recall for all batches of Anaestamine 100mg/ml injection (Vm 41821/4011) as particles have been found in batches of the product and the origin of these particles is unknown.'	[46]
April 2019	United States	Enrofloxacin<br>Ivermectin	4<br>3	Product sterility<br>'There is a concern that if the sterility of these products has been compromised, use of these products could result in introduction of infectious agents to the animal.'	[47]
April 2019	United States	Alfaxalone	2	Impurity defect<br>'This recall has been initiated due to an out of specification result for clarity observed in the stability program. The presence of the aluminum phosphate precipitate presents a potential risk. […] two adverse event reports for lot #25955 and one adverse event report for lot #27787, involving five animals to date.'	[48]

The quality of veterinary medicines and their implications for One Health

May 2019	United Kingdom	Tylosin	1	Product sterility<br>'[…] Elanco UK AH Ltd has issued a recall of Tylan 200mg/ml Solution for Injection (Vm 00006/4098). This is a precautionary measure as sterility cannot be assured.'	[49]
May 2019	United Kingdom	Florfenicol	1	Product sterility<br>'[…] Norbrook Laboratories has issued a recall of Norfenicol 300 mg/ml Solution for injection (Vm 02000/4316) all vial sizes below 250ml as a precautionary measure as sterility cannot be assured.'	[50]
June 2019	Canada	Oxytetracycline	2	Presence of unknown participates<br>'Product: Oxyvet 100 LP Solution. The presence of participates in the affected lots.'	[51]
June 2019	United Kingdom	Alfaxalone	3	Presence of particles of unknown origin<br>'[…] This recall is because particulates have been found in identified batches of product during routine stability testing program.'	[52]
Unspecified 2017 - 2019	France<br>Ukraine<br>Hungary<br>Germany<br>United Kingdom<br>Poland<br>Czech Republic<br>Serbia<br>Spain<br>Netherlands<br>Denmark<br>Croatia<br>Thailand<br>United Arab Emirates<br>Sri Lanka<br>Egypt	Rabies vaccine<br>Sulfamonomethoxine<br>Tetrametrin<br>Tylosin<br>Cefalonium<br>Inactivated duck parvovirus<br>Newcastle disease virus<br>Colistin<br>Amoxicillin<br>Tylvalosin<br>Atipamezole<br>Oxytocin<br>Paravox ovis virus<br>Ampicillin-Cloxacillin<br>Procaine-Epinephrine	3<br>1<br>1<br>1<br>1<br>1<br><br>1<br>1<br>1<br>1<br>1<br>1<br>1<br>1<br>1	Substandard and falsified veterinary product reports received by WHO – no details on defects	[53]

The quality of veterinary medicines and their implications for One Health

	Cuba<br>Barbados<br>Jamaica	Turkey herpes virus<br>Carprofen<br>Oxytetracycline<br>Tilmicosin<br>Meloxicam<br>Ketamine<br>Enrofloxacin<br>Ivermectin<br>Abamectin<br>Bronopol	1<br>1<br>1<br>1<br>1<br>1<br>1<br>1<br>1<br>1		
August 2019	United Kingdom	Fusidic acid	1	Presence of particles of unknown origin<br>'This recall is due to an out of specification result for particle size being found in routine stability testing.'	[54]
September – October 2019	Canada	Phenylbutazone	6	API content – no details on API content failure<br>'The concentration is out of specification for the affected lots.'	[55,56]
December 2019	India	Albendazole	1	pH failure<br>'Name of Drugs: Albendazole Vet. Oral Suspension (FLUKGON); B. No.: 205, Mfg dt: 11/2018, Exp dt: 10/2020, Mfd by: Albro Pharmaceutcals Pvt. Ltd. GMP Certified Company Muktsar 152026, Punjab.'	[57]
December 2019	Canada	Selamectin	1	Packaging defect<br>'Product: Revolution 120 mg/mL solution. The affected lot may contain a tube of another product (Stronghold Plus 30 mg/5 mg)'	[58]
December 2019	United Kingdom	Amoxicillin-Clavulanic acid-Prednisolone	11	API content – no details on API content failure<br>'Following a recent Good Manufacturing Practice (GMP) inspection of one of the active pharmaceutical ingredient (API) manufacturers for Synulox LC, a statement of non-compliance with GMP has been issued.'	[59]

The quality of veterinary medicines and their implications for One Health

January 2020	Canada	Ivermectin	7	API content - no details on API content failure<br>‘Product: Ivermectin Pour-On for Cattle 5mg/ml solution. The assay is out of specification in the affected lots’	[60]
January 2020	Canada	Ivermectin	3	API content - no details on API content failure<br>‘Product: Bovimectin 5mg/ml solution. The assay is out of specification in the affected lots’	[61]
January 2020	United Kingdom	Amoxicillin-Clavulanic acid	14	Stability concerns<br>‘[…] This recall is because the efficacy of the product cannot be guaranteed after 12 months.’	[62]
February 2020	India	Oxytetracycline	2	Water content failure<br>‘Oxytetracycline Hydrochloride (VET) IP/EP/USP, B. No.: Y51806089W, Mfg dt: 01/06/2018, Exp dt: 31/05/2022, Mfd by: M/s. Hebei Jainmin Starch Glucose Co. Ltd., Industrial Development Zone, Ningjin Country, Hebei, China’<br><br>Bacterial endotoxins test failure<br>‘STECLIN INJECTION Veterinary 50 mg/ml (Oxytetracycline Hydrochloride Injection I.P. 30 ml), B. No.: STIR19113, Mfg dt: 08/2019, Exp dt: 07/2021, Mfg by: M/s. Zydus Animal Health, A Division of Cadila Healthcare Ltd., At. Plot No. 69/1, G.I.D.C. Kansari Khambhat, Dist. Anand 388 630, Gujarat’	[63]
February 2020	Canada	Amoxicillin-Clavulanic acid	3	Mislabeling<br>‘Product:<br>1. Clavamox Chewable 125 mg Tablets<br>2. Clavamox Chewable 250 mg Tablets<br>3. Clavomox Chewable 375 mg Tablets.<br>The dosage indicated on the outer label of the affected lots is incorrect’	[64]
February 2020	Canada	Glycerine-Iodine	1	API content – no details on API content failure	[65]

The quality of veterinary medicines and their implications for One Health

				'Product: LC-MV 10 Liquid. The concentration of medicinal ingredient (iodine) may be out of specification in the affected lot.'	
March & September 2020	United Kingdom	Prednisolone	3	Microbial contamination<br>'[…] This recall is due to microbial contamination'	[66,67]
April 2020	United Kingdom	Toldimfos	1	Product defect<br>'In the course of an internal review, it was noted that the available data for the product no longer meets the current EU regulations for food-producing species. […]'	[68]
September 2020	United Kingdom	Imidacloprid	1	Mislabeling<br>'On the carton leaflet, it states: For the prevention and treatment and prevention of flea (Ctenocephalides felis) infestations on cats of less than 4 kg body weight. It should state: For the prevention and treatment and prevention of flea (Ctenocephalides felis) infestations on cats greater or equal to 4 kg body weight.'	[69]
September 2020	United Kingdom	Carprofen	4	Impurity defect<br>'[…] During stability monitoring analysis of the product an impurity was detected which exceeded the specification for an individual impurity. The impurity was isolated and the best estimate of identity indicated that the impurity is carprofen related.'	[70]
September 2020	United Kingdom	Febendazole	8	API content – Low API and product defect<br>'[…] Febendazole content has been found lower than the agreed specification limit and sediment/caking layer is present on bottom of the bottle. […]'	[71]
January 2021	United Kingdom	Carprofen	7	Impurity defect<br>'[…] This is a precautionary measure due to higher than usual impurities.'	[72]

The quality of veterinary medicines and their implications for One Health

January 2021	Canada	Chlorhexidine acetate	All batches	API content – Incorrect API<br>'Product: Blue Velvet Teat Dip. Affected lots contain incorrect medicinal ingredient (i.e. Chlorhexidine Gluconate instead of Chlorhexidine Acetate)'	[73]
January 2021	Canada	Chlorhexidine acetate	All batches	API content – Incorrect API<br>'Product: COSMONAUT BLUE. Affected lots contain incorrect medicinal ingredient (i.e. Chlorhexidine Gluconate instead of Chlorhexidine Acetate)'	[74]
February 2021	United Kingdom	Chlorocresol-Phenoxyethanol-Zinc oxide-Lidocaine	2	API content – no details on API content failure<br>'[…] Petlife International Ltd has initiated a Class II recall to retail level for the product Otodex Skin Cream as the lidocaine content is marginally out of specification.'	[75]

The quality of veterinary medicines and their implications for One Health
Seizures

Month and year of publication	Country	API/API combination	No. batches	Description of the incident	Reference
April 2004	Ireland	Unspecified	Unstated	Falsified – illegal production<br>'[…] The operation code-named "Nora", was part of a clamp down on the distributers and manufacturers of illegal animal remedies. The raids on farms, homes and businesses involved over 100 garda in the Republic and the same number of PSNI officers in the North, backing up teams from the Departments of Agriculture and Health. A raid in Co Tyrone uncovered an illegal animal drugs manufacturing plant and a large quantity of counterfeit drugs were seized'	[76]
June 2003	India	Oxytetracycline	1	Poor quality – no details on API content failure<br>'The officials of the Drug Control Administration on Wednesday seized certain batches of 16 categories of drugs that were found to be not of standard quality. […] The names of the drugs and its manufacturers, along with batch number in bracket, are: […] Oxytetracycline HCL Animal form tabs, Rac Remedies Pharmaceuticals, Ujjain (90)"	[77]
December 2004	Ireland	Unspecified	Unstated	Falsified<br>'[…] Labels for unauthorized products, thousands of bottles and piles of raw materials to make drugs were all seized.'	[78]
October 2005	India	Sulfamethoxazole<br>Tetracycline	1<br>1	Falsified – no API<br>'APDCA has seized veterinary oral preparation of Biosol Vet of batch number BS 909, manufactured by TQM Healthcare, Chennai. They claimed that the active ingredient, Sulfamethaxazole was not present in these drugs. […] The team seized oral preparation of Tetravin Vet, bearing batch number TVP 099, manufactured by	[79]

The quality of veterinary medicines and their implications for One Health

				Medivin Pharmaceuticals […]. The drug, which claimed to contain tetracycline hydrochloride, was lacking the active ingredient.'	
November 2005	China	Bird flu vaccines	Unstated	Falsified – illegal production<br>'The Ministry of Agriculture has punished 13 domestic institutions involved in the production and sale of fake bird flu vaccines, withdrawing its approval for them to produce vaccines for this disease.'	[80]
January 2007	Burkina Faso	Unspecified	Unstated	Falsified<br>' […], 85 types de faux médicaments vétérinaires d'une valeur totale de 50.000.000 FCFA. Parmi ces produits se trouvent notamment des antibiotiques, des antiparasitaires, des vaccins, et des compléments vitaminiques, provenant du Cameroun, de Sénégal, de France, de Hollande, de Chine et d'Espagne.'	[81]
August 2009	India	Sulphadoxine	Unstated	Poor quality - no details on API content failure<br>'In a random selection of drugs from the pharmacy outlets in the three states it was found that all the three labs have unanimously opined that drugs are not known to contain the requires bulk in adequate quantities. […] The drug that have been found substandard are: […] Sulphaquinoxaline solution for veterinary use […]'	[82]
August 2010	United states	Fipronil	Unstated	Falsified – contained pesticides<br>'John Buerman ran a business called CatsMart Plus via the internet. He sold unregistered, unlabeled pesticides for cats and dogs while infringing on the trademark of two well known national brand names, 'Frontline' and 'Frontline Plus'.' […] 'Testing at EPA's National Enforcement Investigations Center (NEIC) laboratory confirmed that the products contained unregistered pesticides.'	[83]
August 2011	United Kingdom	Unspecified	Unstated	Falsified<br>'[…] Meddes and Lansley had warehouses for spurious medicines imported from unidentified locations in	[84]

The quality of veterinary medicines and their implications for One Health

				Belgium and Kent. They illegally sold non steroidal anti-inflammatory drugs, anabolic steroids, antibiotics, sedatives, and pain control treatments for a variety of species including horses, cows, sheep, pigs and household pets.’	
August 2013	China	Unspecified	Unstated	Falsified<br>‘Eight main suspects, including the company manager, who was identified only by his surname of Xiong, were arrested, and police seized more than 4,000 boxes with 20 kinds of counterfeit drugs. Six kinds of the counterfeit drugs contained clenbuterol.’	[85]
September 2013	China	Unspecified	Unstated	Falsified<br>‘Counterfeit veterinary drugs seized during the focus on the destruction of 2,500 kilograms of counterfeit veterinary drugs from the city's 10 districts and counties of winter green sword action with the role of disinfection, anti-bacterial, antiinflammatory, dermatological treatment 1,129,280 bottles / bags / boxes, the value of amount of 11.2 million.’	[86]
January 2014	India	Levamisole	Unstated	Poor quality – no details on API content failure and impurity test failure<br>‘In the case of South East Pharmaceuticals in SIDCO Industrial Area in Chennai, its product Helmisol Powder or levamisole hydrochloride veterinary oral powder, which was seized by the state drug inspectors from Chitradurga was seen to be not of standard quality with regards to its description as its contained lumps and its content levamisole hydrochloride did not conform it its label claim.’	[87]
July 2014	India	Chlorpheniramine<br>Enrofloxacin	Unstated<br>Unstated	Poor quality – Low API (less than 70%) and stability test<br>‘On July 2, the department went on to issue another list with drugs such as […] Chloraxin injection containing Chlorpheniramine for veterinary use manufactured by Zoetis India at Haridwar, […] Meriquin Vet with is an	[88]

The quality of veterinary medicines and their implications for One Health

				Enrofloxacin injection by Stanex Drugs and Chemicals at Hyderabad […]. The drugs are declared not-of-standard quality when their active ingredients are below 70 per cent […].'	
September 2014	Benin<br>Mozambique<br>Togo	Unspecified	Unstated	Falsified<br>'Pour la première fois, un trafic significatif de produits vétérinaires illicites a été détecté: plus d'un million d'injectables au Bénin, plus d'un million de comprimes et d'ampoules au Mozambique, et plus de 100.000 injectables au Togo.'	[89]
March 2015	Cameroun	Oxytetracycline	Unstated	Falsified<br>'[…] Among other medicines destroyed, we could counted […] injection solutions for animals Oxymed 100ml'	[90]
March 2015	India	Piroxicam	Unstated	Poor quality – no details on API content failure<br>'Karnataka drugs control department has found 51 drugs picked up randomly from various pharmacy outlets not of standard quality […]. They include […], Penof injection for veterinary use manufactured by Embark Lifesciences in Roorkee, […]. At times drug fails quality tests because of poor content of the active pharmaceutical ingredient.'	[91]
August 2015	China	Unspecified	Unstated	Falsified<br>'Twelve people were caught producing and selling fake animal drugs, [...] Police in Jinan, Shandong's capital, also seized more than 7,000 kilograms.'	[92]
September 2015	Pakistan	Unspecified	Unstated	Poor quality and expired veterinary medicines – no details on defect<br>'[…] La *Islamabad Capital Territory administration*, alertée par la découverte de prescriptions, pour des humains, de médicaments à usage vétérinaire, a mené une opération conduisant à la fermeture de treize hôpitaux et l'arrestation de douze « faux médecins ». Le préfet de police Abdul Sattar Isani a précisé que les hôpitaux ont	[93]

The quality of veterinary medicines and their implications for One Health

				été fermes pour cause d'insalubrité et d'utilisation de médicaments sous-standards et périmes.'	
August 2016	India	Meloxicam	Unstated	Poor quality – no details on API content failure<br>'Karnataka drugs control department has seized 11 not of standard quality drugs picked up randomly from pharmacy outlets in different areas of the city. Of the 11 drugs which have been tested […], Zodbid-M which is a veterinary injection containing meloxicam manufactured by Zydus Animal Health […]. The drugs are found to be not of standard quality […]. At times drug fails quality tests because of poor quality of the active pharmaceutical ingredients '	[94]
December 2016	United states	Unspecified	Unstated	Falsified<br>'A Laguna Hills man was arrested Tuesday morning on charges that he used the internet to sell misbranded veterinary medications without a prescription.'	[95]
May 2017	Taiwan	Porcine circovirus vaccine	Unstated	Falsified – Low API<br>'In the investigation into the deaths of piglets in Yunlin and Changhua counties, Yunlin prosecutors said, they found that the animals have been injected with a counterfeit product that was basically a diluted vaccine.'	[96]
June 2017	India	Niclosamide-Albendazole<br>Nimesulide-Paracetamol-Serratiopeptidase	Unstated<br><br>Unstated	Poor quality – Unknown defect<br>'Karnataka drugs control department has seized 16 not of standard quality drugs. […]. The 16 drugs which were found to be not of standard quality are […] Albenic 500ml suspension for veterinary purposes. The drug is manufactured by Vet India Pharmaceuticals contains niclosamide and albendazole. pyridase which contains nimesulide, paracetamol and serratiopepidase bolus manufactured by Pure & Cure Healthcare at Uttarkhand.'	[97]
June 2017	Ireland	Unspecified	Unstated	Falsified<br>'Veterinary drugs were seized by the Police Service of Northern Ireland (PSNI), during a planned search of premises near Crossmaglen in Co Armagh.	[98]

The quality of veterinary medicines and their implications for One Health

				The drugs were not registered for use in NI and were seized as part of an operation carried out by the PSNI in conjunction with the Veterinary Medicines Directorate in Defra.'	
January 2018	India	Oxytocin	Unstated	Falsified<br>'Over 20,000 samples of veterinary oxytocin along with its packaging materials worth about Rs20 lakh were seized from Machhuatoli […] presence of unblistered samples, unavailability of any purchase receipt, sale invoice and raw materials prove that the hormone was meant for illegal sale in retail market. The samples were kept in different cartons while their labels were stored in separate boxes, he said.'	[99]
May 2018	India	Oxytocin	1	Unauthorized<br>'[…] the inspectorate staff of CDSCO, Sub-Zone Jammu has seized Oxytocin Injection B.P.(VET) 1 ML, B.No. 001, D/M: 04/2013, D/E: 04/2015, Manufactured by M/s T.D.M Pharma, Mfg. unit: Khujuria B.Deoghar from a Provisional Store (unlicensed premises).'	[100]
June 2018	Morocco	Unspecified	1	Falsified<br>'One batch of medicines from Asia on sale without authorization was seized'	[101]
July 2018	India	Oxytocin	Unstated	Falsified – Illegal production<br>'The manufacturing licence of Nelko Chemical had expired on August 23, 2017 and the firm did not renew it. 335,500 ampoules of oxytocin worth R 57 lakh in the market were seized from the drug company. The ampoules were being manufactured without blister pack with an intention to divert the stocks for veterinary use. […] Another seizure was made at Anand Pharmaceuticals. 225,200 ampoules of oxytocin worth R 38 lakh were seized from the drug firm as it was found manufacturing oxytocin injections without blister pack with an intention to divert the stocks for veterinary use.	[102]

The quality of veterinary medicines and their implications for One Health

				The manufacturing licence of the company expired on December 31, 2016 and since then it was not renewed.'	
March 2019	United Kingdom	Methylprednisolone	Unstated	Unauthorized<br>'Depo-medrol is an anti-inflammatory human medicine which had been imported from the Netherlands with no valid import certificate.'	[103]
May 2019	United Kingdom	Clostridium	Unstated	Unauthorized<br>'The following product was seized: Tribovax 10 100ml x 12 bottles. This product is a sheep and cattle vaccine which does not hold a marketing authorization in the UK.'	[104]
July 2019	United Kingdom	Fipronil<br>Meloxicam	Unstated<br>Unstated	Unauthorized<br>'The following products were seized as they were suspected to have been obtained unlawfully, or to beyond their use-by date, or both.<br>- 1 x Johnsons Fipronil spot-on solution for cats<br>- 1 x Meloxydil 1.5mg oral suspension for dogs<br>- 1 x Metacam 1.5mg oral suspension for dogs<br>- 1 x Frontline spot on for dogs (Non UK authorized)<br>- 1 x pipette Frontline spot on for dogs'	[105]
August 2019	United Kingdom	Oxytocin	Unstated	Unauthorized<br>'The following products were seized:<br>- 1 x 50ml bottle of oxytocin (part used)<br>- 1 x 100ml bottle of oxytocin (part used)<br>Oxytocin is a hormone treatment used to initiate contractions in various animals, including food producing. These were not UK authorized products. […]'	[106]
September 2019	India	Unspecified	Unstated	Falsified – Illegal production<br>'A man was arrested by Indore police on Wednesday for running an illegal factory making fake medicines to boost buffaloes milk productivity in Dwarakapuri. […] Soon after this, police called drug department for the investigation of medicines found in large quantity in the	[107]

The quality of veterinary medicines and their implications for One Health

				illegal factory. As many as 4,000 bottles of medicines, 16 bottles of phenyl, 3 acidic acid canes, 300 l tanker and packaging materials were seized by the police, […]’	
October 2019	United Kingdom	Dexamethasone	Unstated	Unauthorized<br>‘A parcel was detained and subsequently seized at the Border Force, Stansted Airport. […]. These products intended for use in horse are not authorized products in the UK.’	[108]
October 2019	United Kingdom	Buserelin	Unstated	Unauthorized<br>‘The following unauthorised product was seized following a routine inspection of a veterinary practice: 1 x 10ml Busol 0.004mg/ml (part used). […] This is not a UK authorised version.’	[109]
October 2019	United Kingdom	Chlortetracycline	Unstated	Mr. Yearling received a seizure notice of unauthorized possession of Animedazon Spray, 2.45% w/w.<br>‘Details of seizure notice served to Mr Yearling, Plymouth. The following product was seized: Animedazon Spray, 2.45% w/w. Animedazon is a Prescriptionn Only Medicine – Veterinary (POM-V) […]. Unauthorised possession and administration of POM-V veterinary medicines are offences under regulation 7 (Classification, supply and possession of the product) and regulation 8 (Administration of the product) of the Veterinary Medicines Regulations 2013.’	[110]
November 2019	United Kingdom	Unspecified	Unstated	Unauthorized<br>‘The following products were seized by a VMD inspector during an unannounced visit to the pet shop as they are unauthorized veterinary medicines: 61 x Bob Martin Clear Spot on. The UK authorized version of this product is used for flea treatment for cats and dogs. The seized products were marketed for other European countries and therefore not labelled or authorized for retail supply in the UK.’	[111]
November 2019	United Kingdom	Cannabidiol	Unstated	Unauthorized	[112]

The quality of veterinary medicines and their implications for One Health

				‘The following products were seized by a VMD inspector from a stand at the National Pet Show as they were marketed as veterinary medicines:<br>- 3 x 125ml bottles of CBD oil<br>- 4 x 150g sachet of CBD powder	
January 2020	United Kingdom	Amikacin<br>Tiludronic acid	Unstated	Unauthorized<br>‘The following products were seized by a VMD inspector during a routine inspection of a veterinary practice:<br>- 2 x 50ml Bottle of Amikavet 125mg/ml<br>- 2 Boxes of 10 doses of Tidren 5mg/ml<br>- 1 Box of 10 doses of Tidren 5mg/ml (6 doses remaining)<br>These products are non-UK veterinary products. […]’	[113]
February 2020	United Kingdom	Unspecified	Unstated	Unauthorized<br>‘The following product was seized by a VMD inspector during a routine inspection of a veterinary practice: 4 x 100ml bottles of P-Block. This product is a non-UK veterinary product. […]’	[114]
February 2020	United States	Unspecified	Unstated	Falsified – Illegal production<br>‘A man previously convicted of killing his wife also spent years mixing together random ingredients and selling them to desperate dog owners under the guise that his concoction cured canine cancer, […] Prosecutors allege Nyce used various websites to promote and ship the fake drugs “Tumexal” and “Naturasone”, claiming they cured a ‘wide variety’ of cancer.[…]’	[115]
February 2020	Uganda	Unspecified	Unstated	Falsified – Illegal production<br>‘The suspects were found in possession of counterfeit veterinary drug worth Shillings 150 Million. […] the culprits have been duplicating pesticides for spraying bedbugs, cockroaches and other insects and re-package them as acaricides for spraying animal. Which they rebrand as Tick Burn.’	[116]
June 2020	United Kingdom	Phenylbutazone	Unstated	Unauthorized	[117]

The quality of veterinary medicines and their implications for One Health

		Ketoprofen<br>Doxycycline<br>Oxytocin<br>Unspecified	Unstated<br>Unstated<br>Unstated<br>Unstated	'The following veterinary medicines were detained and subsequently seized at Border Force, Inverness Airport following an attempt to import:<br>- 1 x Equine Bute Paste<br>- 2 x 100ml Ketoprofen Injection<br>- 2 x Buscopan Compositum<br>- 2 x 100ml Doxycycline Paste<br>- 2 x 5ml Calmat Injection<br>- 6 x 50ml Syntocin Injections<br>- 100 x 1.3g sachets of Butalone Granules<br>These products were intended for use in horses and are not UK authorised products'	
October 2020	United Kingdom	Amoxicillin-Clavulanic acid	Unstated	Mr. Michael Dawson received a seizure notice of unauthorized possession of veterinary medicinal products. 'Details of the prosecution of Mr Michael Dawson, trading as Element Bullys Limited, Plymouth.'<br>'The following products were seized by Defra Investigation Services (DIS) as part of an investigation case and under the execution of a search warrant.<br>- 1 box containing several blister strips box labelled Clavubactin 250<br>Clavubactin 250 is a Prescription Only Medicine – Veterinary (POM-V). It is an antibiotic treatment for dogs. Unauthorised possession and administration of POM-V veterinary medicines are offences under Regulation 7 (Classification, supply and possession of the product) and Regulation 8 (Administration of the product) of the VMR.<br>- 2 x 50ml bottles labelled Oxytocin Solution<br>- 1 bottle labelled Synulox<br>- 1 bottle labelled Noradine 24<br>- 1 bottle labelled PG600 Injekcio<br>- 5 vials x 10ml labelled Busol<br>These products are non-UK veterinary product.'	[118,119]

The quality of veterinary medicines and their implications for One Health

October 2020	United Kingdom	Chlortetracycline<br>Pyrantel-Febantel-Praziquantel<br>Buserelin<br>Dexamethasone<br>Canine herpes vaccine<br>Fusidic acid<br>Marbofloxacin<br>Canine distemper vaccine-Canine parvovirus vaccine<br>Acepromazine<br>Amitraz<br>Unspecified	Unstated<br>Unstated<br>Unstated<br>Unstated<br>Unstated<br>Unstated<br>Unstated<br>Unstated<br>Unstated<br>Unstated<br>Unstated	Susan Bello-Pearson received a seizure notice of unauthorized possession of veterinary medicinal product. 'Details of the seizure notice served to Susan Bello-Pearson, Dezinerbullz Ltd, Essex in January 2018. The following products were seized by Defra Investigation Services (DIS) as part of an investigation case and under the execution of a search warrant:<br>- 1 box labelled Aureomycin 10mg/g<br>- 7 boxes labelled Aniprantel Tabletta<br>- 3 tablets in a pack labelled Aniprantel<br>- 2 open 10ml vials labelled Busol<br>- 6 x 10ml vials labelled Busol<br>- 1 box labelled Dexafort<br>- 10 Sachets labelled Doxacin 100mg/g<br>- 1 empty blister pack labelled Erythromycin<br>- 1 box labelled Enroxil 5%<br>- 1 empty 1ml vial labelled Eurican Herpes 205<br>- 1 opened 1ml vial labelled Eurican Herpes 205<br>- 6 x 1ml vials labelled Eurican Herpes 205<br>- 10 tablets, 9 in a blister pack and 1 loose, all labelled Flagyl 200mg<br>- 2 box labelled Isathal eye drops<br>- 12 tablets in a blister pack labelled Metronidazole<br>- 14 tablets in 2 blister packs labelled Metronidazole<br>- 1 open bottle labelled Marbocyl 10<br>- 1 blister labelled Noroclav 250mg tablets for dogs<br>- 1 box labelled Nobivac Diluent A.U.V.<br>- 3 boxes containing a total of 23 x 1ml vials labelled Nobivac DP Vaccine A.U.V.<br>- 1 box containing 8 x 1ml vials labelled Nobivac DP Vaccine A.U.V.<br>- 3 boxes containing a total of 28 x 1ml vials labelled Nobivac Diluent A.U.V.	[120–122]

The quality of veterinary medicines and their implications for One Health

				- 3 boxes labelled Oculsan 5ml<br>- 9 boxes labelled Surolan<br>- 1 used syringe labelled Sedalin 35mg/ml<br>- 2 boxes labelled Sedalin<br>- 1 plastic bottle labelled Taktic<br>- 1 pack containing 3 vials (1 used)<br>- 5 blister packs of 20 tablets<br>- 1 pack containing 3 vials (1 used)<br>- 1 open bottle for injectable use with a worn label<br>These products are non-UK veterinary product.'<br><br>'Details of the seizure notice served to Susan Bello-Pearson, Dezinerbullz Ltd, Chelmsford, Essex in April 2018. The following products were seized by Defra Investigation Services (DIS) as part of an investigation case and under the execution of a search warrant.<br>- Blister pack containing 10 tablets labelled Synulox 250mg – not on SN<br>- Blister pack containing 9 tablets labelled Ronaxan 100mg<br>- Blister pack with 10 empty cells labelled Ronaxan 100mg<br>- 3 boxes labelled Noroclav 250mg tablets for dogs<br>- Blister pack containing 4 tablets labelled Noroclav 250mg for Dogs<br>- Blister pack containing 4 tablets labelled Noroclav 500mg for Dogs<br>The medicines listed above are all Prescription Only Medicine – Veterinary (POM-V). They are all antibiotic treatments for dogs.'	
November 2020	United Kingdom	Oxytocin<br>Unspecified	Unstated<br>Unstated	Unauthorized<br>'This parcel was addressed to a residential premise in the UK and contained:<br>- 1 bottle of Oxytocin 100 ml Injection	[123,124]

The quality of veterinary medicines and their implications for One Health

				This product is a non-UK veterinary medicine. It is labelled for use in both food producing and companion animals. The UK authorised version of this product is a hormone with indications including increasing contractions during labour.'<br><br>'This parcel was addressed to a residential premise in the UK and contained:<br>- 2 boxes of 10 x 5ml vials of Gestavet<br>- 2 boxes of 10 x 5ml vials of Solvent<br>These products are non-UK veterinary medicines. The UK authorized version of this product is intended for use in pigs and indications include induction and synchronization of heat.'	
November 2020	United Kingdom	Omeprazole	Unstated	Unauthorized<br>'This parcel was addressed to a residential premises in the UK and contained:<br>- 30 x AbGard, Omeprazole Oral Paste 37%w/w<br>- 10 sachets of AbPrazole Plus x<br>These products are intended for use in horses and are not authorised veterinary medicines in the UK.'	[125]
December 2020	United Kingdom	Omeprazole	Unstated	Unauthorized<br>'This parcel was addressed to residential premises in Strabane, County Tyrone and was shipped from India. The parcel contained:<br>- 4 boxes, each containing 18 syringes, of Ashwazole, omeprazole oral paste 37%w/w<br>This product is intended for use in horses and is not authorised veterinary medicine in the UK.'	[126]
January 2021	United Kingdom	Unspecified	Unstated	Unauthorized<br>'This parcel was addressed to residential premises in Banbridge, County Down and was shipped from South Africa. The parcel contained:	[127]

The quality of veterinary medicines and their implications for One Health

				- 2 x 1kg bags of Fosbac Plus T water soluble broad spectrum antibiotic compound<br>This product is labelled for the treatment of bacterial infection in poultry and swine. It is not authorized as a veterinary medicine in the UK.'	

The quality of veterinary medicines and their implications for One Health
Case reports

Month and year of publication	Country	API/API combination	No. batches	Description of the incident	Reference
September 2005	Nigeria	Diminazen-Phenazone	4	Poor quality – Low API<br>Two out of four preparation containing less than the stated active compound.<br>One batch of 'Berenil' contained 94.1% of diminazen.<br>One batch of 'Dophanil' contained 89.8% of diminazen.	[128]
April 2008	United States	Rabies vaccine	Unstated	'During the reporting period, the CVB investigated 4 reports of lack of efficacy in dogs where rabies was suspected as result of a positive test. Of these, 2 dogs were confirmed as rabid by the CDC and 2 were not. […]. For the other dog confirmed positive for rabies, the serial vaccine of the last rabies product used was still within the period before the expiration date. Testing indicated that the product no longer met minimum required potency, therefore, the lot of this serial vaccine was subsequently recalled from the market.'	[129]

The quality of veterinary medicines and their implications for One Health
REFERENCES:

1 Vision Reporter. Fake veterinary drugs on the market. New Vis. Uganda's Lead. Dly. 2005.https://www.newvision.co.ug/new_vision/news/1131986/fake-veterinary-drugs-market (accessed 12 Jul 2019).

2 Health Canada. Co-op (April 11, 2006). 2006.https://healthycanadians.gc.ca/recall-alert-rappel-avis/hc-sc/2006/9365r-eng.php (accessed 26 Feb 2021).

3 Health Canada. Doxirobe Gel (December 20, 2006). 2007.https://healthycanadians.gc.ca/recall-alert-rappel-avis/hc-sc/2006/9527r-eng.php (accessed 26 Feb 2021).

4 PigProgress. Fake veterinary drugs found in China. 2007.https://www.pigprogress.net/Home/General/2007/6/Fake-veterinary-drugs-found-in-China-PP000701W/ (accessed 25 Feb 2019).

5 Health Canada. Neo Sulfalyte Bolus (June 05, 2008). 2008.https://www.healthycanadians.gc.ca/recall-alert-rappel-avis/hc-sc/2008/9680r-eng.php (accessed 26 Feb 2021).

6 Health Canada. Penicillin G Procaine 300,000 IU (October 05, 2009). 2009.https://healthycanadians.gc.ca/recall-alert-rappel-avis/hc-sc/2009/9737r-eng.php (accessed 26 Feb 2021).

7 Keith C. Major veterinary drug recall gets bigger / Animal anesthetic drug pulled off market , but questions remain. SFGATE. 2010.https://www.sfgate.com/pets/yourwholepet/article/Major-veterinary-drug-recall-gets-bigger-Animal-2483151.php (accessed 17 Jun 2019).

8 Health Canada. Topagen Spray (July 28, 2011). 2011.https://healthycanadians.gc.ca/recall-alert-rappel-avis/hc-sc/2011/10207r-eng.php (accessed 26 Feb 2021).

9 Health Canada. Bioclav (November 30, 2011). 2011.https://healthycanadians.gc.ca/recall-alert-rappel-avis/hc-sc/2011/10275r-eng.php (accessed 26 Feb 2021).

10 Health Canada. BNP (October 30, 2012). 2012.https://www.healthycanadians.gc.ca/recall-alert-rappel-avis/hc-sc/2012/15866r-eng.php (accessed 26 Feb 2021).

11 Health Canada. Imbryn Antiseptic Dairy Cream. 2012.https://maliactu.net/113-millions-de-faux-medicaments-saisis-dans-quatorze-pays-africains/ (accessed 23 Jun 2016).

12 Health Canada. Chloramphenicol 1% (2013-11-14). 2013.https://healthycanadians.gc.ca/recall-alert-rappel-avis/hc-sc/2013/37039r-eng.php (accessed 26 Feb 2021).

The quality of veterinary medicines and their implications for One Health

13 Health Canada. BMD 110g (2014-03-10). 2014.https://healthycanadians.gc.ca/recall-alert-rappel-avis/hc-sc/2014/38403r-eng.php (accessed 26 Feb 2021).

14 Case P. Warning issued about fake vet med Metacam. 2014.https://www.fwi.co.uk/livestock/warning-issued-about-fake-vet-med-metacam (accessed 12 Nov 2015).

15 Health Canada. Folltropin-V (2015-04-09). 2015.https://healthycanadians.gc.ca/recall-alert-rappel-avis/hc-sc/2015/52957r-eng.php (accessed 26 Feb 2021).

16 CDSCO. List of Drugs, Medical Devices and Cosmetics declared as Not of Standard Quality/Spurious/Adulterated/Misbranded for the Month of October-2015. Cent. Drugs Stand. Control Organ. CDSCO, India. 2015.https://cdsco.gov.in/opencms/opencms/system/modules/CDSCO.WEB/elements/download_file_division.jsp?num_id=MjM2NQ== (accessed 26 Feb 2021).

17 CDSCO. List of Drugs, Medical Devices and Cosmetics declared as not of standard quality/spurious/adulterated/misbranded. Cent. Drugs Stand. Control Organ. CDSCO, India. 2015.https://cdsco.gov.in/opencms/opencms/system/modules/CDSCO.WEB/elements/download_file_division.jsp?num_id=MjM3MA== (accessed 26 Feb 2021).

18 CDSCO. List of Drugs, Medical Devices and Cosmetics declared as Not of Standard Quality/Spurious/Adulterated/Misbranded for the Month of Decmeber-2015. Cent. Drugs Stand. Control Organ. CDSCO, India. 2016.http://weekly.cnbnews.com/news/article.html?no=124000 (accessed 26 Feb 2021).

19 Health Canada. Onycin 250 (2016-01-21). 2016.https://www.healthycanadians.gc.ca/recall-alert-rappel-avis/hc-sc/2016/56962r-eng.php (accessed 26 Feb 2021).

20 GOV.UK. Vetflurane liquid 250ml – Product defect alert. 2020.https://www.gov.uk/government/news/vetflurane-liquid-250ml-product-defect-alert (accessed 25 Jun 2020).

21 CDSCO. List of Drugs, Medical Devices and Cosmetics declared as Not of Standard Quality/Spurious/Adulterated/Misbranded, For the Month of May, 2016. Cent. Drugs Stand. Control Organ. CDSCO, India. 2016.https://cdsco.gov.in/opencms/opencms/system/modules/CDSCO.WEB/elements/download_file_division.jsp?num_id=MjI3OQ== (accessed 26 Feb 2021).

22 GOV.UK. Hipnoton 10 mg/ml Solution for Injection for Horses and Cattle: Product defect recall alert. 2016.https://www.gov.uk/government/news/hipnoton-10-mgml-solution-for-injection-for-horses-and-cattle-product-defect-recall-alert (accessed 25 Jun 2020).

The quality of veterinary medicines and their implications for One Health

23 GOV.UK. Loxicom 1.5 mg/ml Oral Suspension for Dogs – Product defect recall alert. 2016.https://www.gov.uk/government/news/loxicom-15-mgml-oral-suspension-for-dogs-product-defect-recall-alert (accessed 25 Jun 2020).

24 GOV.UK. Combinex Oral Suspension – Product defect recall alert. 2017.https://www.gov.uk/government/news/combinex-oral-suspension-product-defect-recall-alert (accessed 25 Jun 2020).

25 GOV.UK. Propofol Emulsion for Injection 1.0% w/v - Product defect recall alert. 2017.https://www.gov.uk/government/news/propofol-emulsion-for-injection-10-wv-product-defect-recall-alert (accessed 25 Jun 2020).

26 USFDA. Inc. Issues Voluntary Nationwide Recall of Amitriptyline HCL Tablets, USP 50mg and Phenobarbital Tablets, USP 15mg, 30mg, 60mg, 100mg Due to Potential Label Mix-Up. 2017.https://www.fda.gov/safety/recalls-market-withdrawals-safety-alerts/co-truxton-inc-issues-voluntary-nationwide-recall-amitriptyline-hcl-tablets-usp-50mg-and (accessed 28 May 2019).

27 GOV.UK. Deosect 5 % w / v Concentrate for Cutaneous Spray Solution 250ml – Product Defect Recall Alert. 2017.https://www.gov.uk/government/news/deosect-5-wv-concentrate-for-cutaneous-spray-solution-250ml-product-defect-recall-alert (accessed 25 Jun 2020).

28 GOV.UK. Flypor 4% w/v Pour-on Solution – Product defect recall alert. 2017.https://www.gov.uk/government/news/flypor-4-wv-pour-on-solution-product-defect-recall-alert (accessed 25 Jun 2020).

29 CDSCO. List of Drugs, Medical Devices and Cosmetics declared as Not of Standard Quality/Spurious/Adulterated/Misbranded for the Month of JULY 2015. Cent. Drugs Stand. Control Organ. CDSCO, India. 2017.https://cdsco.gov.in/opencms/opencms/system/modules/CDSCO.WEB/elements/download_file_division.jsp?num_id=MjM2OA== (accessed 26 Feb 2021).

30 GOV.UK. Meloxaid 5mg/ml Solution for Injection for Dogs and Cats – Product defect recall alert. 2017.https://www.gov.uk/government/news/meloxaid-5mgml-solution-for-injection-for-dogs-and-cats-product-defect-recall-alert (accessed 25 Jun 2020).

31 GOV.UK. Ornicure 150mg/g doxycycline powder for oral solution – Product defect recall alert. 2018.https://www.gov.uk/government/news/ornicure-150mgg-doxycycline-powder-for-oral-solution-product-defect-recall-alert (accessed 25 Jun 2020).

32 GOV.UK. Oxytobel 10 IU/ml Solution for Injection for Horses, Cattle, Pigs, Sheep, Goats, Dogs and Cats – Product defect recall alert. 2018.https://www.gov.uk/government/news/oxytobel-10-iuml-solution-for-injection-for-horses-cattle-pigs-sheep-goats-dogs-and-cats-product-defect-recall-alert (accessed 25 Jun 2020).

33 GOV.UK. Alvegesic vet. 10 mg/ml Solution for injection for Horses, Dogs and Cats - Product defect recall alert.

The quality of veterinary medicines and their implications for One Health

2018.https://www.gov.uk/government/news/alvegesic-vet-10-mgml-solution-for-injection-for-horses-dogs-and-cats-product-defect-recall-alert (accessed 25 Jun 2020).

34 GOV.UK. Stresnil 40 mg/ml Solution for Injection for Pigs - Product defect recall alert. 2018.https://www.gov.uk/government/news/stresnil-40-mgml-solution-for-injection-for-pigs-product-defect-recall-alert (accessed 25 Jun 2020).

35 Health Canada. PropoVet Multidose 10mg/ml (2018-06-18). 2018.https://healthycanadians.gc.ca/recall-alert-rappel-avis/hc-sc/2018/67504r-eng.php (accessed 26 Feb 2021).

36 GOV.UK. Crovect 1.25% w/v Pour-on Solution for Sheep - Product defect recall alert. 2018.https://www.gov.uk/government/news/crovect-125-wv-pour-on-solution-for-sheep-product-defect-recall-alert (accessed 25 Jun 2020).

37 GOV.UK. Trimax Plus 4 in 1 liquid for racing pigeons - Product defect recall alert. 2018.https://www.gov.uk/government/news/trimax-plus-4-in-1-liquid-for-racing-pigeons-product-defect-recall-alert (accessed 25 Jun 2020).

38 GOV.UK. Filavac VHD K C+V - Product defect recall alert. 2018.https://www.gov.uk/government/news/filavac-vhd-k-cv-product-defect-recall-alert (accessed 25 Jun 2020).

39 GOV.UK. Willcain Solution for Injection - Product defect recall alert. 2018.https://www.gov.uk/government/news/willcain-solution-for-injection-product-defect-recall-alert (accessed 25 Jun 2020).

40 Ntirenganya E. Govt mounts crackdown on counterfeit livestock drugs. New Times. 2018;:1–2.https://www.newtimes.co.rw/news/govt-ivestock-drugs (accessed 14 Dec 2018).

41 Health Canada. Kaolin Pectin Suspension (2018-08-11). 2018.https://healthycanadians.gc.ca/recall-alert-rappel-avis/hc-sc/2018/67534r-eng.php (accessed 26 Feb 2021).

42 GOV.UK. Carprieve Small Animal Injection – Product defect recall alert. 2018.https://www.gov.uk/government/news/carprieve-small-animal-injection-product-defect-recall-alert (accessed 25 Jun 2020).

43 GOV.UK. Equimax Oral Gel for Horses Yardpacks - Product defect recall alert. 2019.https://www.gov.uk/government/news/equimax-oral-gel-for-horses-yardpacks-product-defect-recall-alert (accessed 25 Jun 2020).

44 USFDA. Stokes Healthcare Inc. Issues Voluntary Nationwide Recall of Pilocarpine 0.1% Ophthalmic Solution Due to a High Level of Preservative | FDA. 2019.https://www.fda.gov/safety/recalls-market-withdrawals-safety-alerts/stokes-healthcare-inc-issues-voluntary-nationwide-recall-pilocarpine-01-ophthalmic-solution-due-high (accessed 20 Mar 2019).

45 USFDA. Norbrook Laboratories Limited Recalls Veterinary Products for Health Risk. 2019.https://www.fda.gov/safety/recalls-market-

withdrawals-safety-alerts/norbrook-laboratories-limited-recalls-veterinary-products-health-risk (accessed 11 Mar 2019).

46 GOV.UK. Anaestamine 100mg/ml injection - Product defect recall alert. 2019.https://www.gov.uk/government/news/anaestamine-100mgml-injection-product-defect-recall-alert (accessed 23 Jun 2020).

47 USFDA. Norbrook Laboratories Limited Expands Recall of Veterinary Products for Health Risk. 2019.https://www.fda.gov/safety/recalls-market-withdrawals-safety-alerts/norbrook-laboratories-limited-expands-recall-veterinary-products?utm_campaign=Norbrook Laboratories Limited Expands Recall of Veterinary Products&utm_medium=email&utm_source=Eloqua (accessed 27 May 2019).

48 USFDA. Jurox Incorporated is Voluntarily Recalling Two Lots of Alfaxan Unpreserved , an Intravenous Injectable Anaesthetic. 2019.https://www.fda.gov/safety/recalls-market-withdrawals-safety-alerts/jurox-incorporated-voluntarily-recalling-two-lots-alfaxan-unpreserved-intravenous-injectable (accessed 28 May 2019).

49 GOV.UK. Tylan 200mg/ml Solution for Injection - Product defect recall alert. 2019.https://www.gov.uk/government/news/tylan-200mgml-solution-for-injection-product-defect-recall-alert (accessed 23 Jun 2020).

50 GOV.UK. Norfenicol 300mg/ml Solution for Injection - Product defect recall alert. 2019.https://www.gov.uk/government/news/norfenicol-300mgml-solution-for-injection-product-defect-recall-alert (accessed 23 Jun 2020).

51 Health Canada. Oxyvet 100 LP (2019-06-04). 2019.https://healthycanadians.gc.ca/recall-alert-rappel-avis/hc-sc/2018/68538r-eng.php (accessed 26 Feb 2021).

52 GOV.UK. Alfaxan & Alfaxan Multidose 10mg/ml Solution for. 2019.https://www.gov.uk/government/news/alfaxan-alfaxan-multidose-10mgml-solution-for-injection-product-defect-recall-alert (accessed 23 Jun 2020).

53 World Health Organization. Substandard and Falsified Medical Products. 2019.https://rr-africa.oie.int/wp-content/uploads/2019/07/lee.pdf (accessed 6 Jan 2021).

54 GOV.UK. Isathal 10 mg/g eye drops, suspension for dogs, cats and rabbits - product defect recall alert. 2019.https://www.gov.uk/government/news/isathal-10-mgg-eye-drops-suspension-for-dogs-cats-and-rabbits-product-defect-recall-alert (accessed 25 Jun 2020).

55 Health Canada. Phenylbutazone Powder (2019-09-23). 2019.https://healthycanadians.gc.ca/recall-alert-rappel-avis/hc-sc/2019/71067r-eng.php (accessed 26 Feb 2021).

56 Health Canada. Phenylbutazone Powder (2019-10-09). 2019.https://healthycanadians.gc.ca/recall-alert-rappel-avis/hc-sc/2019/71303r-eng.php (accessed 26 Feb 2021).

57 CDSCO. List of Drugs, Medical Devices and Cosmetics declared as Not of Standard Quality/Spurious/Adulterated/Misbranded, for the

The quality of veterinary medicines and their implications for One Health

Month of November 2019. Cent. Drugs Stand. Control Organ. CDSCO, India. 2019.https://cdsco.gov.in/opencms/resources/UploadCDSCOWeb/2018/UploadAlertsFiles/drugaleernov2019.pdf (accessed 26 Feb 2021).

58 Health Canada. Revolution (2019-12-12). 2019.https://www.healthycanadians.gc.ca/recall-alert-rappel-avis/hc-sc/2019/72295r-eng.php (accessed 26 Feb 2021).

59 GOV.UK. Synulox Lactating Cow Intramammary Suspension - Product defect recall alert. 2019.https://www.gov.uk/government/news/synulox-lactating-cow-intramammary-suspension-product-defect-recall-alert (accessed 23 Jun 2020).

60 Health Canada. Ivermectin Liquid (2020-01-20). 2020.https://www.healthycanadians.gc.ca/recall-alert-rappel-avis/hc-sc/2020/72297r-eng.php (accessed 26 Feb 2021).

61 Health Canada. Bovimectin (2020-01-21). 2020.https://www.healthycanadians.gc.ca/recall-alert-rappel-avis/hc-sc/2020/72299r-eng.php (accessed 26 Feb 2021).

62 GOV.UK. Product defect recall alert Clavubactin 50/12.5mg, 250/62.5mg, 500/125mg. 2020.https://www.gov.uk/government/news/product-defect-recall-alert-clavubactin-50125mg-250625mg-500125mg (accessed 23 Jun 2020).

63 CDSCO. List of Drugs, Medical Devices and Cosmetics declared as Not of Standard Quality/Spurious/Adulterated/Misbranded, for the Month of February 2020. Cent. Drugs Stand. Control Organ. CDSCO, India. 2020.http://mpoc.org.my/malaysian-palm-oil-industry/ (accessed 26 Feb 2021).

64 Health Canada. Zoetis Canada Clavamox product recall (2020-02-21). 2020.https://healthycanadians.gc.ca/recall-alert-rappel-avis/hc-sc/2020/72453r-eng.php (accessed 26 Feb 2021).

65 Health Canada. LC-MV 10 (2020-02-25). 2020.https://healthycanadians.gc.ca/recall-alert-rappel-avis/hc-sc/2020/72515r-eng.php (accessed 26 Feb 2021).

66 GOV.UK. Prednidale 5mg Tablets - Product defect recall alert. 2020.https://www.gov.uk/government/news/prednidale-5-mg-tablets-product-defect-recall-alert (accessed 23 Jun 2020).

67 GOV.UK. Prednidale 5 mg Tablets - Product defect recall alert 2. 2020.https://www.gov.uk/government/news/prednidale-5-mg-tablets-product-defect-recall-alert-2 (accessed 25 Feb 2021).

68 GOV.UK. Foston 20% w/v Solution for Injection - Product defect recall alert. 2020.https://www.gov.uk/government/news/foston-20-wv-solution-for-injection-product-defect-recall-alert (accessed 25 Jun 2020).

The quality of veterinary medicines and their implications for One Health

69 GOV.UK. Johnson ' s 4Fleas 80mg Spot-on Solution for Cats - Product defect recall alert. 2020.https://www.gov.uk/government/news/johnsons-4fleas-80mg-spot-on-solution-for-cats-product-defect-recall-alert (accessed 25 Feb 2021).

70 GOV.UK. Carprieve 50 mg/ml Solution for Injection for Cattle - Product defect recall alert. 2020.https://www.gov.uk/government/news/carprieve-50-mgml-solution-for-injection-for-cattle-product-defect-recall-alert (accessed 25 Feb 2021).

71 GOV.UK. Gallifen 200 mg/ml Suspension, Pigfen 200 mg/ml Suspension - Product defect recall alert. 2020.https://www.gov.uk/government/news/gallifen-200-mgml-suspension-pigfen-200-mgml-suspension-product-defect-recall-alert (accessed 25 Feb 2021).

72 GOV.UK. Carprieve 5% w/v Small Animal Solution for Injection - Product defect recall alert. 2021.https://www.gov.uk/government/news/carprieve-5-wv-small-animal-solution-for-injection-product-defect-recall-alert (accessed 25 Feb 2021).

73 Health Canada. Blue Velvet Teat Dip (2021-01-22). 2021.https://healthycanadians.gc.ca/recall-alert-rappel-avis/hc-sc/2021/74899r-eng.php (accessed 26 Feb 2021).

74 Health Canada. Cosmonaut blue (2021-01-22). 2021.https://healthycanadians.gc.ca/recall-alert-rappel-avis/hc-sc/2021/74897r-eng.php (accessed 26 Feb 2021).

75 GOV.UK. Otodex Skin Cream - Product defect recall alert. 2021.https://www.gov.uk/government/news/otodex-skin-cream-product-defect-recall-alert (accessed 25 Feb 2021).

76 Macconnell S. Gardai seize illegal animal drugs ; Growing trade in counterfeit veterinary medicines on both sides of the Border. The Irish Times. 2004.http://www.highbeam.com/doc/1P2-24741645.html (accessed 18 Mar 2014).

77 SEARPharm. Database on the incidents of Counterfeit Medicines in the WHO-SEA Region. 2004.

78 Erwin A. Counterfeit vet drugs seized in Border operation. Irish Exam. 2004.https://www.irishexaminer.com/archives/2004/1223/ireland/counterfeit-vet-drugs-seized-in-border-operation-46635447.html (accessed 20 Sep 2013).

79 Pharmabiz.com. APDCA seizes stocks of substandard veterinary drugs in fresh raids. 2005.http://www.pharmabiz.com/NewsDetails.aspx?aid=30034&sid=2 (accessed 25 Mar 2019).

80 Xinhua News Agency. 13 Fake Bird Flu Vaccine-makers Punished. 2005.http://arabic.china.org.cn/english/government/148014.htm

(accessed 25 Oct 2019).

81 Fondation Chirac. Burkina faso : saisie majeure de faux médicaments à bobo-dioulasso. 2007.https://www.fondationchirac.eu/2017/01/burkina-faso-saisie-majeure-de-faux-medicaments-a-bobo-dioulasso/ (accessed 28 Mar 2019).

82 Vijay N. Six commonly used drugs found substandard. 2009.http://www.pharmabiz.com/NewsDetails.aspx?aid=52024&sid=2 (accessed 26 Mar 2019).

83 USEPA. Summary of Criminal Prosecutions. 2010.https://cfpub.epa.gov/compliance/criminal_prosecution/index.cfm?action=3&prosecution_summary_id=2468 (accessed 4 May 2018).

84 Imber S. Giant UK Fake Veterinary Pharmacy Shut Down: Impacts Food Supply Says Authorities. Safemedicines.org. 2011.http://www.safemedicines.org/2011/08/giant-illegal-veterinary-pharmacy-shut-down-impacts-food-supply-says-authorities-305.html (accessed 17 Jun 2019).

85 The Pig Site. Banned substance found in Vet drugs. 2013.https://thepigsite.com/news/2013/08/banned-substance-found-in-vet-drugs-1 (accessed 12 Jul 2019).

86 Drugs Taoyuan Veterinary. Set off around the feed, veterinary drugs crackdown Storm. 2013.http://en.gxtysy.com/news_detail/newsId=52.html (accessed 27 Nov 2015).

87 Vijay N. Karnataka DC dept seizes 7 drugs failing to clear dissolution test & sold with faulty labels. 2014.http://www.pharmabiz.com/NewsDetails.aspx?aid=79930&sid=1 (accessed 26 Mar 2019).

88 Pharmabiz.com. Karnataka DC dept seizes 20 not-of-std quality drugs from pharmacies , cos asked to withdraw stocks. 2014.http://www.pharmabiz.com/NewsDetails.aspx?aid=82889&sid=1 (accessed 26 Mar 2019).

89 MaliActu.net. 113 millions de faux mé dicaments saisis dans quatoze pays africains. 2016.https://maliactu.net/113-millions-de-faux-medicaments-saisis-dans-quatorze-pays-africains/ (accessed 23 Jun 2016).

90 Dongo P. Cameroun - Traffic de médicaments : 35 millions FCFA de médicaments contrefaits détruits dans la ville de Bafoussam. Cameroon-Info.net. 2015.http://www.cameroon-info.net/article/cameroun-traffic-de-medicaments-35-millions-fcfa-de-medicaments-contrefaits-detruits-dans-la-ville-241500.html (accessed 4 Apr 2016).

91 Vijay N. Karnataka DC dept seizes 51 not-of-standard qlty drugs ; cautions hospitals , public to not to use. Pharmabiz.com. 2015.http://www.pharmabiz.com/NewsDetails.aspx?aid=87224&sid=1 (accessed 26 Mar 2019).

The quality of veterinary medicines and their implications for One Health

92 ShanghaiDaily.com. Twelve caught in east China for faking animal drugs. 2015.https://archive.shine.cn/article/article_xinhua.aspx?id=298736 (accessed 9 Oct 2015).

93 Fondation Chirac. Douze « faux médecins » arrêtés et treize hôpitaux fermés au pakistan. 2015.https://www.fondationchirac.eu/2015/09/douze-faux-medecins-arretes-et-treize-hopitaux-fermes-au-pakistan/ (accessed 14 Jun 2016).

94 Vijay N. Karnataka DC seizes 11 NSQ drugs , cautions hospitals , public. Pharmabiz.com. 2016.http://www.pharmabiz.com/NewsDetails.aspx?aid=96963&sid=1 (accessed 26 Mar 2019).

95 USICE. Orange County man charged with selling pet meds without a prescription , some of which were not approved for distribution in the US. 2016.https://www.ice.gov/news/releases/orange-county-man-charged-selling-pet-meds-without-prescription-some-which-were-not (accessed 22 Nov 2019).

96 Focus Taiwan. Two suspects held for allegedly selling fake pig vaccines. 2017.https://www.newvision.co.ug/new_vision/news/1131986/fake-veterinary-drugs-market (accessed 12 Jun 2018).

97 Vijay N. Karnataka drugs control dept detects 16 NSQ drugs , alerts cos and pharmacies. Pharmabiz.com. 2017.http://www.pharmabiz.com/NewsDetails.aspx?aid=102744&sid=1 (accessed 26 Mar 2019).

98 McCann P. Police seize veterinary drugs in Armagh. Irish Farmers J. 2017.https://www.farmersjournal.ie/police-seize-veterinary-drugs-in-armagh-282396 (accessed 19 Nov 2019).

99 Times of India. Veterinary oxytocin worth Rs20 lakh seized. 2018.https://timesofindia.indiatimes.com/city/patna/veterinary-oxytocin-worth-rs20-lakh-seized/articleshow/62385804.cms (accessed 8 Jun 2018).

100 CDSCO. Oxytocin Drug Alert. Cent. Drugs Stand. Control Organ. CDSCO, India. 2018.https://cdsco.gov.in/opencms/opencms/system/modules/CDSCO.WEB/elements/download_file_division.jsp?num_id=MTQ1OQ== (accessed 26 Feb 2021).

101 Alaoui Z. Beni Mellal : Saisie de médicaments vétérinaires de contrebande en vente illicite. 2018.https://www.lereporter.ma/lire-aussi/beni-mellal-saisie-de-medicaments-veterinaires-de-contrebande-en-vente-illicite/ (accessed 26 Apr 2019).

102 Yadav L. CDSCO seizes illegal stock of oxytocin injections worth Rs . 95 lakh from 2 drug companies in Bihar. Pharmabiz.com. 2018.http://www.pharmabiz.com/NewsDetails.aspx?aid=109753&sid=1 (accessed 26 Feb 2019).

103 GOV.UK. Animal medicine seizure notice : Emiliano Espinar Veterinary Surgeon. 2019.https://www.gov.uk/government/news/animal-medicine-seizure-notice-emiliano-espinar-veterinary-surgeon (accessed 23 Jun 2020).

The quality of veterinary medicines and their implications for One Health

104 GOV.UK. Animal Medicine seizure notice : Sallins Farm. 2019.https://www.gov.uk/government/news/animal-medicine-seizure-notice-sallins-farm (accessed 23 Jun 2020).

105 GOV.UK. Animal medicines seizure notice : Mr Thompson. 2019.https://www.gov.uk/government/news/animal-medicines-seizure-notice-mr-thompson (accessed 23 Jun 2020).

106 GOV.UK. Animal medicines seizure notice: Tudor House. 2019.https://www.gov.uk/government/news/animal-medicines-seizure-notice-tudor-house-animal-care-ltd (accessed 23 Jun 2020).

107 Asian News International. Indore: Illegal factory making fake medicines for increasing milk productivity busted, 1 held. 2019.https://www.indiatoday.in/crime/story/indore-illegal-factory-making-fake-medicines-for-increasing-milk-productivity-busted-1-held-1600664-2019-09-19 (accessed 23 Jun 2020).

108 GOV.UK. Animal medicine seizure notice: Border Force, Stansted Airport. 2019.https://www.gov.uk/government/news/animal-medicine-seizure-notice-border-force-stansted-airport (accessed 23 Jun 2020).

109 GOV.UK. Animal medicine seizure notice : Essex Vets. 2019.https://www.gov.uk/government/news/animal-medicine-seizure-notice-essex-vets (accessed 23 Jun 2020).

110 GOV.UK. Animal medicine seizure notice : Mr Yearling. 2019.https://www.gov.uk/government/news/animal-medicine-seizure-notice-mr-yearling (accessed 23 Jun 2020).

111 GOV.UK. Animal medicine seizure notice : Pawfection Pets. 2019.https://www.gov.uk/government/news/animal-medicine-seizure-notice-pawfection-pets (accessed 23 Jun 2020).

112 GOV.UK. Animal medicines seizure notice : NPD Ventures Ltd (T/A World of Pets). 2019.https://www.gov.uk/government/news/animal-medicines-seizure-notice-npd-ventures-ltd-ta-world-of-pets (accessed 23 Jun 2020).

113 GOV.UK. Animal medicine seizure notice : Guasco & Associates. 2020.https://www.gov.uk/government/news/animal-medicine-seizure-notice-guasco-associates (accessed 23 Jun 2020).

114 GOV.UK. Animal medicine seizure notice : Gansera Leveque. 2020.https://www.gov.uk/government/news/animal-medicine-seizure-notice-gansera-leveque (accessed 23 Jun 2020).

115 Chinchilla R. Man Who Killed Wife Charged With Selling Fake Canine Cancer Medicines. 2020.https://www.nbcphiladelphia.com/news/local/man-who-killed-wife-charged-with-selling-fake-canine-cancer-medicines/2288274/ (accessed 23 Jun 2020).

116 The Independent. Seven arrested over counterfeit veterinary drugs. 2020.https://www.independent.co.ug/seven-arrested-over-counterfeit-

veterinary-drugs/ (accessed 3 Jun 2020).

117 GOV.UK. Animal medicine seizure notice: Border Force, Inverness Airport. 2020.https://www.gov.uk/government/news/animal-medicine-seizure-notice-border-force-inverness-airport (accessed 25 Feb 2021).

118 GOV.UK. Animal medicines seizure notices: Element Bullys Ltd. 2020.https://www.gov.uk/government/news/animal-medicines-seizure-notices-element-bullys-ltd (accessed 25 Feb 2021).

119 GOV.UK. Prosecution of Mr Michael Dawson, trading as Element Bullys Limited, Plymouth. 2020.https://www.gov.uk/government/news/prosecution-of-mr-michael-dawson-trading-as-element-bullys-limited-plymouth (accessed 25 Feb 2021).

120 GOV.UK. Animal medicines seizure notice: Susan Bello- Pearson, Dezinerbullz Ltd (Jan). 2020.https://www.gov.uk/government/news/animal-medicines-seizure-notice-susan-bello-pearson-dezinerbullz-ltd-3 (accessed 25 Feb 2021).

121 GOV.UK. Animal medicines seizure notice: Susan Bello- Pearson, Dezinerbullz Ltd (April). 2020.https://www.gov.uk/government/news/animal-medicines-seizure-notice-susan-bello-pearson-dezinerbullz-ltd-1 (accessed 25 Feb 2021).

122 GOV.UK. Prosecution of Ms Susan Bello-Pearson trading as Dezinerpugz Limited (previously Dezinerbullz), Essex. 2020.https://www.gov.uk/government/news/prosecution-of-ms-susan-bello-pearson-trading-as-dezinerpugz-limited-previously-dezinerbullz-essex (accessed 25 Feb 2021).

123 GOV.UK. Animal medicines seizure notice: Border Force, Heathrow Airport (1). 2020.https://www.gov.uk/government/news/animal-medicines-seizure-notice-border-force-heathrow-airport (accessed 25 Feb 2021).

124 GOV.UK. Animal medicines seizure notice: Border Force, Heathrow Airport (2). 2020.https://www.gov.uk/government/news/animal-medicines-seizure-notice-border-force-heathrow-airport-2 (accessed 25 Feb 2021).

125 GOV.UK. Animal medicines seizure notice: Border Force, East Midlands Airport. 2020.https://www.gov.uk/government/news/animal-medicines-seizure-notice-border-force-east-midlands-airport (accessed 25 Feb 2021).

126 GOV.UK. Animal medicine seizure notice: Parcel addressed to Strabane, County Tyrone. 2020.https://www.gov.uk/government/news/animal-medicine-seizure-notice-parcel-addressed-to-strabane-county-tyrone (accessed 25 Feb 2021).

127 GOV.UK. Animal medicine seizure notice: Parcel addressed to Banbridge, County Down.

2021.https://www.gov.uk/government/news/animal-medicine-seizure-notice-parcel-addressed-to-banbridge-county-down (accessed 25 Feb 2021).

128 Anene BM, Ezeokonkwo RC, Mmesirionye TI, *et al.* A diminazene-resistant strain of Trypanosoma brucei brucei isolated from a dog is cross-resistant to pentamidine in experimentally infected albino rats. *Parasitology* 2006;**132**:127–33. doi:10.1017/S0031182005008760

129 Frana TS, Clough NE, Gatewood DM, *et al.* Postmarketing surveillance of rabies vaccines. *J Am Vet Med Assoc* 2008;**232**:1000–2. doi:10.2460/javma.232.7.1000
